# Evaluation of biomarkers for in vitro prediction of drug-induced nephrotoxicity: comparison of HK-2, immortalized human proximal tubule epithelial, and primary cultures of human proximal tubular cells

**DOI:** 10.1002/prp2.148

**Published:** 2015-05-15

**Authors:** Johnny X Huang, Geraldine Kaeslin, Max V Ranall, Mark A Blaskovich, Bernd Becker, Mark S Butler, Melissa H Little, Lawrence H Lash, Matthew A Cooper

**Affiliations:** 1 Institute for Molecular Bioscience, The University of Queensland306 Carmody Road, St Lucia, Queensland, 4072, Australia; 2 Department of Pharmacology, School of Medicine, Wayne State University540 East Canfield Avenue, Detroit, Michigan, 48201

**Keywords:** Biomarker, kidney, KIM-1, M-CSF, nephrotoxicity, NGAL

## Abstract

There has been intensive effort to identify in vivo biomarkers that can be used to monitor drug-induced kidney damage and identify injury before significant impairment occurs. Kidney injury molecule-1 (KIM-1), neutrophil gelatinase-associated lipocalin (NGAL), and human macrophage colony stimulating factor (M-CSF) have been validated as urinary and plasma clinical biomarkers predictive of acute and chronic kidney injury and disease. Similar validation of a high throughput in vitro assay predictive of nephrotoxicity could potentially be implemented early in drug discovery lead optimization to reduce attrition at later stages of drug development. To assess these known in vivo biomarkers for their potential for in vitro screening of drug-induced nephrotoxicity, we selected a panel of nephrotoxic agents and examined their effects on the overexpression of nephrotoxicity biomarkers in immortalized (HK-2) and primary (commercially available and freshly in-house produced) human renal proximal tubule epithelial cells. Traditional cytotoxicity was contrasted with expression levels of KIM-1, NGAL, and M-CSF assessed using ELISA and real-time quantitative reverse transcription PCR. Traditional cytotoxicity assays and biomarker assays using HK-2 cells were both unsuitable for prediction of nephrotoxicity. However, increases in protein levels of KIM-1 and NGAL in primary cells were well correlated with dose levels of known nephrotoxic compounds, with limited correlation seen in M-CSF protein and mRNA levels. These results suggest that profiling compounds against primary cells with monitoring of biomarker protein levels may have potential as in vitro predictive assays of drug-induced nephrotoxicity.

## Introduction

Nephrotoxicity, or damage to the kidney, is a serious side effect of many marketed drugs, with 19–25% of acute renal failures caused, at least in part, by drug exposure (Bonventre et al. 2010; McCullough et al. 2013). In the search for new drug therapies, a simple and high-throughput in vitro screen to detect the potential for new drug candidates to cause nephrotoxicity would be invaluable. Human kidney damage is currently identified by in vivo monitoring of serum creatinine (SCr) or blood urea nitrogen (BUN) levels, but correlation to injury is poor (Bonventre et al. 2010). There has been an intensive effort in recent years to identify in vivo biomarkers that can be used to selectively monitor kidney damage at an earlier stage (Dieterle et al. 2010a; Hoffmann et al. 2010; Ozer et al. 2010; Sistare et al. 2010; Huang et al. 2014). In particular, proteins such as kidney injury molecule-1 (KIM-1/TIM-1/HAVCR-1) (Hoffmann et al. 2010; Vaidya et al. 2010), neutrophil gelatinase-associated lipocalin (NGAL/LCN2) (Borkham-Kamphorst et al. 2011; Paragas et al. 2011), and human macrophage colony-stimulating factor 1 (M-CSF/CSF-1) (Wada et al. 1997; Isbel et al. 2001; Menke et al. 2009) have been highlighted as proteins with clinical relevance to early detection of kidney injury. Moreover, the U.S. Food and Drug Administration (FDA) and European Medicines Agency (EMEA) have qualified seven renal safety biomarkers including KIM-1 for use in drug development (Dieterle et al. 2010b).

During preclinical drug development, the gold standard assay for nephrotoxicity potential is kidney histopathology following in vivo administration to animals; a low throughput and expensive method. As a consequence, these studies are generally conducted at a later stage of preclinical drug discovery, by which time considerable resources have already been expended identifying and optimizing the lead compound(s). This workflow does not allow for optimization of toxicity-driven medicinal chemistry during the initial lead optimization phase, when many compounds are produced and screened. SCr, BUN, or other in vivo biomarker levels can be monitored as an alternative to histopathology, but they still require animal testing. A predictive in vitro cell-based assay would be of great value for drug discovery programs, as screening and structure–activity relationship studies could be conducted at a reasonable cost, much earlier in the process. Such an assay could counter-screen multiple drug candidates for kidney toxicity, allowing for simultaneous optimization of on-target potency and reduction of off-target effects. For example, the automated patch clamp human ether-a-go-go related gene (hERG) ion channel assay is now accepted as an early predictor for potential cardiotoxicity and is used routinely at an early stage in the drug discovery process (Priest et al. 2008). Currently, in vitro screening for nephrotoxicity is still based on measuring cell death (White and Seaman 1995), which is indicative of nonspecific cytotoxicity rather than the injury or stress specific to kidney cells. Furthermore, the reliability of these in vitro systems to reproduce the effects observed in a functioning kidney remains uncertain. In particular, there has been very limited study into the potential for predictive in vitro utility for biomarkers that are gaining recognition as clinical surrogates (Huang et al. 2014). A number of in vivo biomarkers have been identified in human studies, but have not yet been translated to early stage screening assays (Huang et al. 2014).

Two commercially available human kidney cell types, human kidney 2 (HK-2) and hRPTECs (primary human renal proximal tubule epithelial cells) were first evaluated in our study. The HK-2 cells are immortalized and have been widely used for renal toxicity studies (Zhipeng et al. 2006; Sung et al. 2008; Wu et al. 2009; Gunness et al. 2010); whereas hRPTECs are primary cells isolated from human kidney proximal tubules (Glynne 2000; Rudnicki et al. 2007). We also prepared fresh renal proximal tubule epithelial cells (hPT) from human kidneys (Lash et al. 2008) and investigated biomarker performance in these cells. None of the above cell types have been extensively evaluated for the expression of kidney injury biomarkers after nephrotoxin treatment.

In the present study, we first evaluated a set of known nephrotoxic drugs, including colistin, gentamicin, cisplatin, cyclosporin A (CsA), amphotericin B (AmB), and doxorubicin (Sabra and Branch 1990; Falagas and Kasiakou 2006; Pabla and Dong 2008; Miller et al. 2010; Lopez-Novoa et al. 2011) using widely used cytotoxicity assays, and then investigated the potential application of in vivo biomarkers (KIM-1, NGAL, and M-CSF) for predicting drug-induced nephrotoxicity at sub-cytotoxic concentrations using in vitro cell culture approaches, assessing multiple concentrations of nephrotoxins at multiple time points. To our knowledge, this is the first systematic study investigating in vivo kidney injury biomarkers in both immortalized and primary cells, using multiple nephrotoxic compounds.

## Materials and Methods

### Compounds

Colistin sulfate (C4461), gentamicin sulfate (G1914), cisplatin (P4394), CsA (30024), AmB (A4888), and doxorubicin (D1515) were purchased from Sigma-Aldrich (Sydney, NSW, Australia).

### Isolation and culture of hPT cells

hPT cells were derived from whole human kidneys procured by the International Institute for the Advancement of Medicine (Edison, NJ). All tissues were scored by a pathologist as normal (i.e., derived from noncancerous, nondiseased tissue). Cell isolation procedures were based on those originally described by Todd et al. (1996) and modified (Cummings and Lash, 2000; Cummings et al., 2000) with use of sterile conditions (i.e., all instruments and glassware were autoclaved and all buffers were filtered through a 0.2-*μ*m pore-size filter). Renal cortex and outer stripe were cut into slices, washed with sterile phosphate buffered saline (PBS), minced, and the pieces were placed in a trypsinization flask filled with 300 mL of sterile, filtered Hanks’ buffer, containing 25 mmol/L NaHCO_3_, 25 mmol/L 4-(2-hydroxyethyl)-1-piperazineethanesulfonic acid (HEPES), pH 7.4, 0.5 mmol/L ethylene glycol tetraacetic acid (EGTA), 0.2% (w/v) bovine serum albumin, 50 *μ*g/mL gentamicin, 1.3 mg/mL collagenase, and 0.59 mg/mL CaCl_2_, which was filtered prior to use. Whole kidneys were perfused with Wisconsin medium and kept on ice until they arrived at the laboratory, which was usually within 24 h of removal from the donor.

All buffers were continuously bubbled with 95% O_2_/5% CO_2_ and were maintained at 37°C. Minced cortical pieces from whole kidneys were subjected to collagenase digestion for 60 min, after which the supernatant was filtered through a 70-*μ*m mesh filter to remove tissue fragments, centrifuged at 150*g* for 7 min, and the pellet resuspended in Dulbecco’s Modified Eagle’s Medium: Ham’s F12 Medium (DMEM/F12; 1/1). Approximately, 5–7 × 10^6^ cells were obtained per 1 g of human kidney cortical tissue.

hPT cells were resuspended in 2 mL of DMEM/F12 and diluted to 500 mL with cell culture medium, which was serum free and hormonally defined. Composition of this supplemented medium was based on earlier work establishing optimal conditions for primary culture of rat PT cells (Lash et al., 1995). Basal medium was a 1:1 mixture of DMEM/F12. Standard supplements included 15 mmol/L HEPES, pH 7.4, 20 mmol/L NaHCO_3_, antibiotics for day 0 through day 3 only (192 IU penicillin G/mL + 200 *μ*g streptomycin sulfate/mL or 50 *μ*g gentamicin/mL) to inhibit bacterial growth, 2.5 *μ*g amphotericin B/mL to inhibit fungal growth, 5 *μ*g bovine insulin/mL (= 0.87 *μ*mol/L), 5 *μ*g human transferrin/mL (= 66 nmol/L), 30 nmol/L sodium selenite, 100 ng hydrocortisone/mL (= 0.28 *μ*mol/L), 100 ng epidermal growth factor/mL (= 17 nmol/L), and 7.5 pg 3,3′,5-triiodo-dl-thyronine/mL (= 111 nmol/L). Cells were seeded in a volume of 0.5 mL at a density of 50–100 *μ*g protein per cm^2^ (0.5–1.0 × 10^6^ cells/mL) on 24-well plates. Cultures were grown at 37°C in a humidified incubator under an atmosphere of 95% air/5% CO_2_ at pH 7.4. Cultures were grown to approximately 80–90% confluence (generally 5–6 days) prior to experiments. Cells were harvested by either scraping the flasks with a Teflon scraper or by brief incubation with Cellstripper (Cellgro, Herndon, VA) (in Ca^2+^- and Mg^2+^-free Hanks’ buffer).

### Cell culture and drug treatment procedures

HK-2 cells (ATCC CRL-2190; Manassas, VA) were maintained in DMEM/F12 (Life Technologies; Sydney, NSW, Australia) supplemented with 5 *μ*g/mL insulin, 5 *μ*g/mL transferrin, 5 ng/mL sodium selenite (ITS; Roche; Sydney, NSW, Australia), 100 U/mL penicillin, 100 g/mL streptomycin, 0.1 *μ*mol/L hydrocortisone, 2 nmol/L l-glutamine plus 10% fetal bovine serum (FBS, Lonza, Sydney, NSW, Australia). hRPTECs (passage 2) and culture kits were purchased from Lonza. hRPTECs were maintained in Renal Epithelial Cell BulletKit™ (CC-3190, Lonza) at 37°C and 5% CO_2_ atmosphere. Cells were passaged to 96-well plates with a density of 4000 cells/well. After 24 h culture, compounds with different concentration series were added into each well and incubated for 4, 24, 48, and 72 h.

### Cytotoxicity assays

MTT and resazurin assays were performed as previously described with slightly modifications (McMillian et al. 2002; van Meerloo et al. 2011). In brief, after a 72 h incubation with compounds, 0.5 mg/mL 3-(4,5-dimethylthiazol-2-yl)-2,5-diphenyltetrazolium bromide (MTT, Life Technologies) or 5 *μ*mol/L resuzurin (Sigma-Aldrich) was added to each well and incubated for 2 h at 37°C, 5% CO_2_. For the MTT assay, the medium was then removed, the dried crystals were resuspended in 60 *μ*L of Dimethyl sulfoxide (DMSO), and the absorbance was read at 570 nm. For the resazurin assay, the fluorescence intensity was read using a PolarStar Omega plate reader (BMG Labtech; Mornington VIC, Australia) at excitation/emission 560/590 nm. All assays were performed at least three times. CC_50_ values were determined using GraphPad Prism® 6 software (Inc. La Jolla, CA). Both MTT and resazurin assays were performed three times, with two technical replicates in each assay.

### ELISA assay

KIM-1 (DY1750), NGAL (DY1757), and M-CSF (DY216) Duo-Set® ELISA kits were purchased from R&D systems (Minneapolis, MN). Sandwich-ELISA experiments were conducted following the manufacturer’s instructions with slight modifications. A 96-well microplate (MaxiSorp®, Nunc, Thermo Scientific, NSW, Australia) was coated with 100 *μ*L of capture antibodies (diluted 1:200 in PBS) and incubated overnight. The plate was then washed twice with washing buffer (0.05% Tween 20 in PBS, pH 7.4) and blocked by adding 300 *μ*L of blocking buffer (1% bovine serum albumin (BSA) in PBS) for 1 h at room temperature. After washing two times with PBS, 100 *μ*L of samples (cell culture media or cell lysates) or standards were then added to each well and incubated for 2 h at room temperature. The biotinylated detection antibodies were then diluted in blocking buffer, added to each well and incubated for 2 h. Horseradish peroxidase (HRP)-labeled streptavidin (100 *μ*L) was then added to bind to detection antibodies. To each well, 100 *μ*L of substrate solution (1:1 mixture of H_2_O_2_ and tetramethylbenzidine) was added and incubated for 20 min before reaction termination with 50 *μ*L of stop solution (2N H_2_SO_4_). The optical density of each well was measured at 450 nm using a POLARstar Omega plate reader (BMG Labtech). All assays were performed at least three times, with at least two technical replicates in each assay.

### Total RNA isolation and real-time PCR

HK-2 and hRPTECs were treated with compounds for 4, 24, 48, and 72 h, then the cells were lysed and the RNA reverse transcribed with the TaqMan® Gene Expression Cells-to-CT™ Kit (Life Technologies) according to the manufacturer’s instructions. For reverse transcription PCR the following TaqMan® Gene Expression Assay probes were used: hypoxanthine phosphoribosyltransferase 1 (*Hprt1*) (Hs01003267_m1), *Havcr1* (KIM-1; Hs00273334_m1), *Lcn2* (NGAL; Hs01008571_m1), and *Csf1* (M-CSF; Hs00174164_m1). All probes span an exon–exon junction. The real-time quantitative reverse transcription PCR (qRT-PCR) was performed in a 10 *μ*l final reaction mixture using a ViiA 7™ system (Applied Biosystems®, Sydney, NSW, Australia) based on the following thermo cycling conditions: 10 min at 95°C for one cycle, 15 sec at 95°C, 60 sec at 60°C for 40 cycles. The ViiA 7™ software was used for data analysis with *Hprt1* expression used for normalization. All assays were performed at least three times, with at least two technical replicates in each assay.

### Statistical analysis

Statistical analysis was performed using GraphPad Prism® 6 software. The groups with clearly dose-dependent responses were analyzed by one-way analysis of variance (ANOVA) test, compared with the lowest concentration group of each nephrotoxicant. For all the tests, *P < *0.05 was considered as a statistically significant difference.

## Results

### Traditional cytotoxicity assays are not reliable indicators for nephrotoxicity screening

To investigate the reliability of traditional cytotoxicity assays in predicting drug-induced nephrotoxicity, MTT, and resazurin assays were performed using both HK-2 cells and hRPTECs. The cells were treated with compounds for 72 h and the CC_50_ (the concentration of 50% cellular toxicity) calculated (Table1). Most of the compounds were not cytotoxic in the MTT assay at the highest concentrations tested, except for cisplatin, which had a CC_50_ value of 75 *μ*mol/L against HK-2 cells. The resazurin assay was more sensitive than the MTT assay, with cisplatin, CsA, AmB, and doxorubicin having measurable CC_50_ values at the concentrations tested (Table1). However, gentamicin and CsA still had no toxicity at the highest concentrations tested, which were 1 mmol/L and 50 *μ*mol/L, respectively. The HK-2 cells and hRPTECs gave broadly similar results.

**Table 1:** Cytotoxicity of compounds in traditional assays in HK-2 cells and hRPTECs

Compounds	CC_50_ (*μ*mol/L)			
	MTT assay		Resazurin assay	
	HK-2	hRPTECs	HK-2	hRPTECs
Colistin	>300	>300	>300	177 ± 15
Gentamicin	>1000	>1000	>1000	>1000
Cisplatin	75 ± 10	>100	15 ± 2	48 ± 2
CsA	>50	>50	>50	>50
AmB	>50	>50	20 ± 3	6 ± 1
Doxorubicin	>50	>50	2.1 ± 1	5.2 ± 1

CC_50_ values were shown as Mean ± Standard deviation, *n *= 3.

### Expression of biomarkers in HK-2 cells

**Figure 1:**
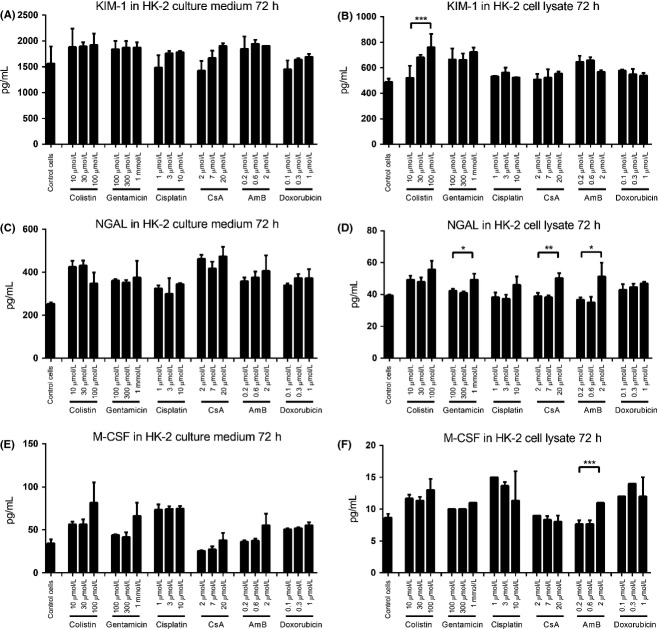
Expression profile of biomarkers in HK-2 cells after nephrotoxic compound treatment for 72 h. (A) KIM-1 protein concentration in culture medium; (B) KIM-1 protein concentration in cell lysates; (C) NGAL protein concentration in culture medium; (D) NGAL protein concentration in cell lysates; (E) M-CSF protein concentration in culture medium; and (F) M-CSF protein concentration in cell lysates. Data are presented as Mean ± Standard deviation. Significantly different *P < 0.05; **P < 0.01; ***P < 0.005, *n *≥ 3 (three independent assays with two technical replicates in each assay).

We first investigated the effects of the six nephrotoxic compounds on the protein levels of KIM-1, NGAL and M-CSF using a quantitative ELISA kit. HK-2 cells were cultured in 96-well plates and incubated with three concentrations (high, medium, and low) of each compound. The high concentrations used for inducing biomarker expression were varied for each compound so that they were lower than the respective CC_50_, in order to induce cell injury but reduce the possibility of nonspecific toxic events, while the low concentration was 10-fold less. Both culture medium and cell lysates were assessed after 4, 24, 48 (Figs. S1–S3), and 72 h (Fig.1) exposure to the compounds. Limited biomarker overexpression was observed compared to the control groups, and there was no significant dose-dependent increase at the 4 h time point (Fig. S1). An AmB–induced M-CSF increase in HK-2 cell lysate was observed at 24 h (Fig. S2F); however, the result may not be reliable as it was reaching the detection sensitivity limitation of the ELISA kits (around 10 pg/mL). After 48 h, CsA induced an increase in NGAL, while gentamicin and AmB induced increases in M-CSF in culture media (Fig. S3C and E). Nevertheless, dose-dependent overexpression became less obvious at 72 h in culture medium (Fig.1A, C, and E). Biomarker overexpression was observed in cell lysates for some compounds at 72 h, but was not consistent for all compounds or biomarkers (Fig.1B, D, and F).

**Figure 2:**
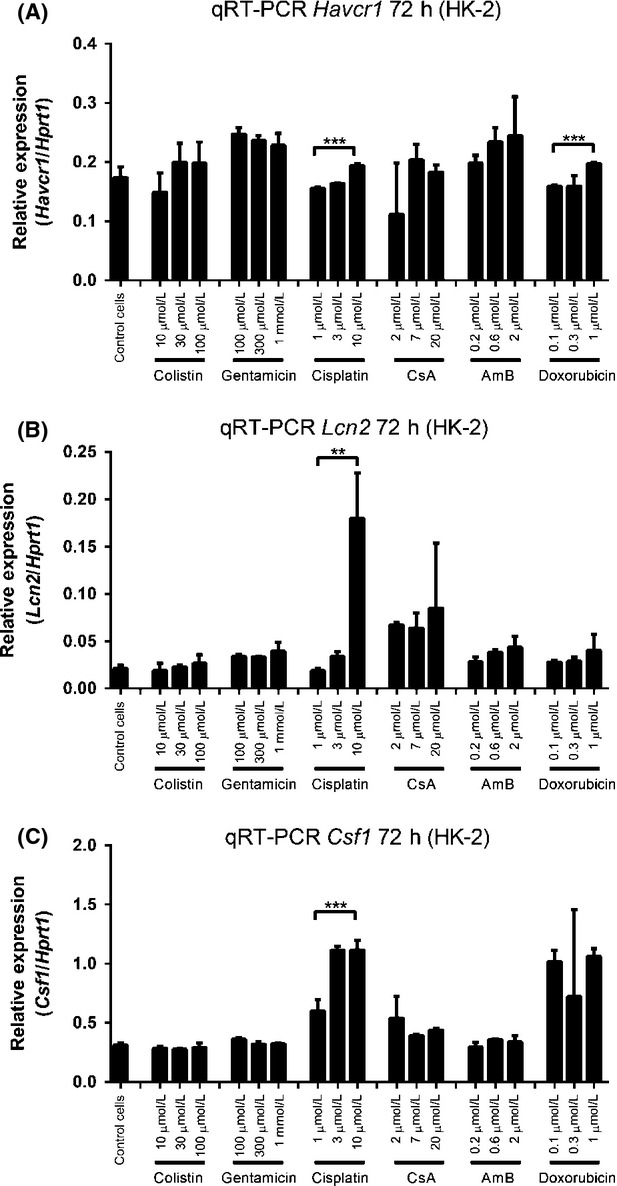
mRNA levels of each biomarkers in HK-2 cells after nephrotoxic compound treatment for 72 h. (A) KIM-1 mRNA, (B) NGAL mRNA and (C) M-CSF mRNA. Data are presented as Mean ± Standard deviation. Significantly different **P < 0.01; ***P < 0.005, *n *≥ 3 (three independent assays with two technical replicates in each assay).

We next evaluated the mRNA levels of each biomarker at the 72-h time point using qRT-PCR (Fig.2). The transcription level of KIM-1 mRNA was increased by cisplatin and doxorubicin treatment (Fig.2A, *P* *<* 0.005), while the mRNA levels of NGAL (Fig.2B, *P* *<* 0.01) and M-CSF (Fig.1C, *P* *<* 0.005) were clearly increased after 72 h exposure to cisplatin, but not other nephrotoxins. Other time points (4, 24, 48 h) of qRT-PCR results were shown in Figure S4. The increased mRNA levels observed did not correlate with increased protein expression. These data indicated that the HK-2 cells are not suitable as a general tool for in vitro biomarker prediction of drug-induced nephrotoxicity.

### Expression of biomarkers in primary cells

We next repeated the same set of experiments using primary hRPTECs, which are phenotypically more representative of renal proximal tubules (Wieser et al. 2008). As observed with HK-2 cells, low doses of the nephrotoxic drugs did not induce significant upregulation of any the biomarkers at 4 h (Fig. S5), except for AmB-induced KIM-1 (Fig. S5A) and colistin-induced NGAL (Fig. S5C).

**Figure 3:**
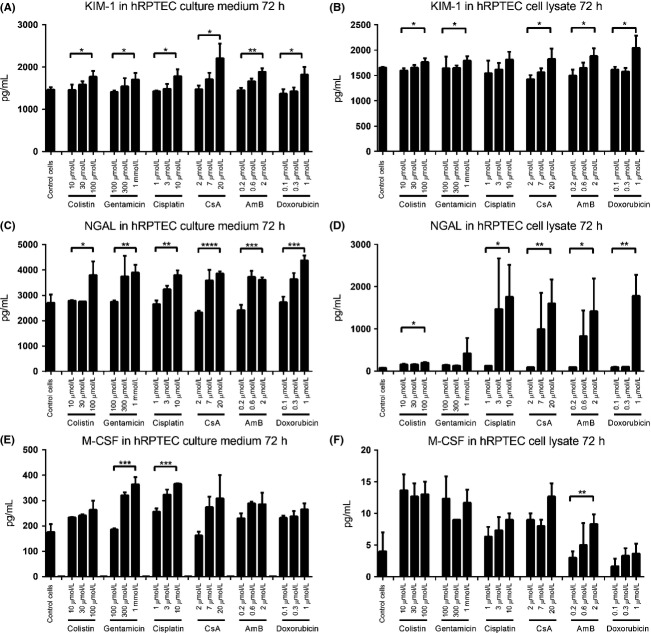
Expression profile of biomarkers in hRPTEC cells after nephrotoxic compound treatment for 72 h. (A) KIM-1 protein concentration in culture medium; (B) KIM-1 protein concentration in cell lysates; (C) NGAL protein concentration in culture medium; (D) NGAL protein concentration in cell lysates; (E) M-CSF protein concentration in culture medium; and (F) M-CSF protein concentration in cell lysates. Data are presented as Mean ± Standard deviation. Significantly different *P < 0.05; **P < 0.01; ***P < 0.005, ****P < 0.001, *n *≥ 3 (three independent assays with two technical replicates in each assay).

Intriguingly, the results from 24 h showed different expression profiles for each biomarker. KIM-1 protein levels were increased in the culture medium by all six nephrotoxins (Fig. S6A), with some compounds also causing increases in NGAL (induced by AmB and doxorubicin, Fig. S6C). Similar expression patterns were also observed in cell lysates (Fig. S6B, D, and F). A more obvious dose-dependent overexpression of the biomarkers was observed at 48 h (Fig. S7). Much more significant dose-dependent responses of each biomarker were observed after 72 h of compound treatment (Fig.3). KIM-1, NGAL, and M-CSF protein release into culture medium was induced by medium and high concentrations of each compound and were dose dependent, although some increases in M-CSF were not statistically significant (Fig.3A, C, and E). KIM-1 protein levels in cell lysates showed similar increases as those in the culture medium (Fig.3B). Strikingly, NGAL protein expressing in cell lysates was increased up to 20-fold compared to the control cells and low-dose treatment (Fig.3D). The increase in biomarker expression over time is illustrated for one compound (Fig. S9).

**Figure 4:**
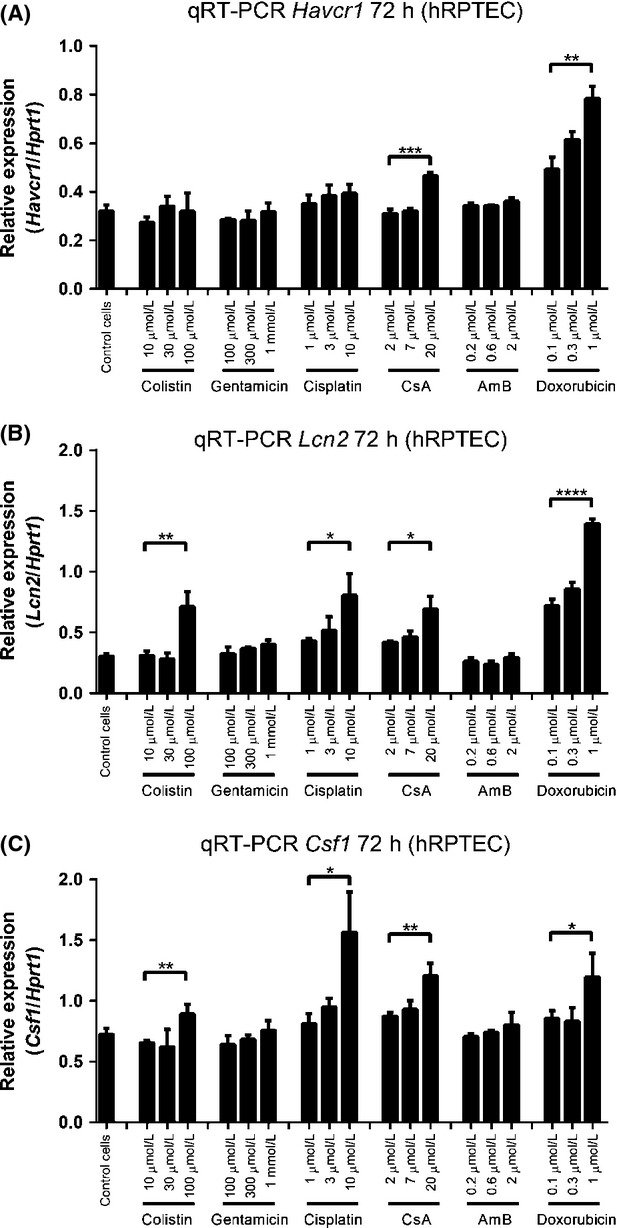
mRNA levels of each biomarkers in hRPTEC cells after nephrotoxic compound treatment for 72 h. (A) KIM-1 mRNA, (B) NGAL mRNA, and (C) M-CSF mRNA. Data are presented as Mean ± Standard deviation. Significantly different *P < 0.05; **P < 0.01; ***P < 0.005; ****P < 0.001, *n *≥ 3 (three independent assays with two technical replicates in each assay).

The mRNA levels of each biomarker were again evaluated (Fig.4). KIM-1 mRNA did not show stimulation upon compound treatment, except for high-dose CsA and doxorubicin at 72 h (Fig.4A), while mRNA levels of NGAL showed a dose-dependent increase at 48 h treatment (Fig. S8F) and kept rising after 72 h exposure (Fig.4) to 100 *μ*mol/L colistin (*P < *0.01), 10 *μ*mol/L cisplatin (*P < *0.05), 20 *μ*mol/L CsA (*P < *0.05), and 1 *μ*mol/L doxorubicin (*P < *0.001) (Fig.4B). In addition, M-CSF mRNA levels displayed a similar pattern to NGAL mRNA, giving significant results in colistin, cisplatin, CsA, and doxorubicin treatment (Fig.4C). Other time points of mRNA expression levels are shown in Figure S8. These more widespread increases in mRNA expression were consistent with the greater increases in protein levels seen for multiple nephrotoxins in hRPTECs at 72 h, although the protein analysis provided more statistically significant increases.

**Figure 5:**
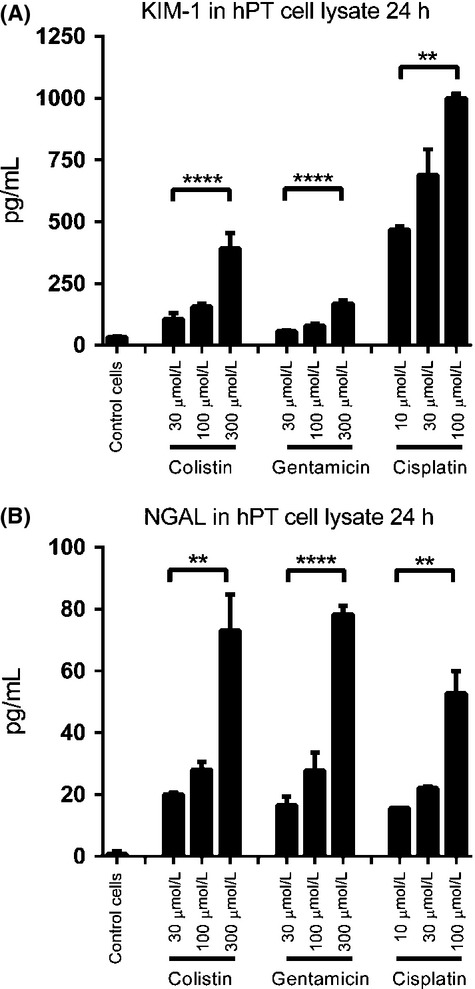
Expression profile of biomarkers in hPT cells after nephrotoxic compound treatment for 24 h. (A) KIM-1 protein concentration in cell lysate, and (B) NGAL protein concentration in cell lysates. Data are presented as Mean ± Standard deviation. Significantly different **P < 0.01; ****P < 0.001, *n *≥ 3 (three independent assays with two technical replicates in each assay).

To further confirm the results, we isolated fresh primary kidney epithelial (hPT) cells from a human kidney and investigated the expression of biomarkers upon nephrotoxin treatment. Due to limited resources, we only investigated KIM-1 and NGAL protein levels in cell lysate after 24 h exposure to high doses of colistin, gentamicin, and cisplatin. The performance of both biomarkers was excellent, showing clear dose-dependent responses and significant increases in protein levels (Fig.5). KIM-1 protein increased 12-fold after 24 h treatment with 300 *μ*mol/L colistin, fivefold after treatment with 300 *μ*mol/L gentamicin, and 32-fold after treatment with 100 *μ*mol/L cisplatin (Fig.5A). NGAL protein showed even better sensitivity, exhibiting more than 60-fold increase for all three compounds at the highest concentrations (Fig.5B). This higher sensitivity was consistent with the greater statistical significance observed in the results in the hRPTECs.

## Discussion

Nephrotoxicity is a significant adverse effect of many marketed drugs. There is an urgent need to develop a more robust, cost-effective, and rapid screening system to detect potential nephrotoxicity at an early stage of drug discovery and development (Huang et al. 2014). We assessed the predictive capabilities of traditional cytotoxic assays in both immortalized and primary kidney proximal tubule epithelial cells, then examined the potential for in vivo kidney injury biomarkers to predict drug-induced nephrotoxicity in vitro.

Assays that measure indicators of general cellular cytotoxicity, such as the MTT and resazurin assays, have been widely used over the past two decades. These assays are mainly focused on assessing metabolic activity and cell death rather than cellular injury. In the present study, we measured the CC_50_ values of well-known nephrotoxic drugs on HK-2 cells and hRPTECs (Table1). Almost all tested nephrotoxins failed to show significant toxicity against both cell types at the highest tested concentration in the MTT assay. The resazurin assay was more sensitive; however, it was not able to reflect the injury to the cell at lower concentrations that are likely to be clinically relevant. Our cytotoxicity results are similar to those reported previously in HK-2 cells (Zager et al. 2007; Wu et al. 2009), while the published data regarding drug-induced toxicity on hRPTECs are very limited (Benesic et al. 2005; Vidal et al. 2006). In general, the cytotoxicity assays were inaccurate for in vitro nephrotoxicity prediction.

Next-generation biomarkers have recently been reported as reliable means to detect drug-induced kidney toxicity in vivo (Bonventre et al. 2010; Dieterle et al. 2010a,b), but there have been few attempts to transfer these novel kidney injury biomarkers to in vitro cellular systems for drug-induced nephrotoxicity screening (Huang et al. 2014). Rached and colleagues investigated the expression of kidney injury biomarkers in rat proximal tubule cells (NRK-52E) (Rached et al. 2008), but found that they were not suitable. A recent report suggested HK-2 cells could be used to evaluate cisplatin-induced nephrotoxicity using western blot and reverse transcription PCR analysis (Sohn et al. 2013). Intriguingly, we were not able to fully reproduce their results.

In this study we evaluated changes in expression of three biomarkers (KIM-1, NGAL, and M-CSF) in three types of human kidney cells. KIM-1 is considered to be a sensitive, specific, and accurate method for the prediction of human nephrotoxicity induced by nephrotoxic drugs (Han et al. 2002; Vaidya et al. 2006; Dieterle et al. 2010b; Ozer et al. 2010). After injury, the ectodomain is shed from proximal tubular epithelial cells into urine, and urinary KIM-1 increases over 100-fold (Vaidya et al. 2010). KIM-1 has been implicated in the pathogenesis of hepatitis (Silberstein et al. 2009), as a modulator to enhance virus–receptor interactions (Tami et al. 2007), and involved in the regeneration of renal cells (Bailly et al. 2002). The upregulation of KIM-1 is also known to result in interstitial fibrosis (Humphreys et al. 2013). NGAL is part of the lipocalin superfamily and is involved in regulating immune responses, transporting iron and modulating cell growth and metabolism (Flo et al. 2004; Schmidt-Ott et al. 2007). Both NGAL protein and mRNA are significantly upregulated after kidney injury, making it a useful and novel biomarker (Mishra et al. 2003; Paragas et al. 2011). M-CSF is a cytokine predominantly generated by kidney tubular epithelial cells (Isbel et al. 2001; Lenda et al. 2003; Menke et al. 2009) that plays an important role in proliferation and differentiation of hematopoietic precursor cells. During injury, the expression level of M-CSF in the kidney and circulation increase in parallel with progressive renal damage (Wada et al. 1997), which renders it an indicator of acute kidney injury. Recent research showed that M-CSF is involved in kidney growth and repair by encouraging the endogenous renal macrophages to take on an M2 response (Alikhan et al. 2011).

The concentrations of each compounds used for inducing biomarker expression were carefully selected, aiming to induce cell injury but minimize nonspecific toxicity. In addition, in vivo plasma concentrations of each compound were also considered and tested in our in vitro assays. For example, the plasma concentration of cisplatin is reported as 1–3 *μ*g/mL (3–10 *μ*mol/L) after 90 min administration (Hanada et al. 2001), which has been covered in our assays.

Our data clearly demonstrate that immortalized HK-2 cells are not suitable as an in vitro nephrotoxicity screening system. Protein and mRNA levels of each biomarker were unreliably upregulated in HK-2 cells after nephrotoxin treatment, with only scattered significant dose-dependent responses observed after 48 and 72 h treatment. Biomarkers in HK-2 cells are not consistently predictive of known nephrotoxic compounds. The transcription profile of HK-2 cells treated with 5 *μ*mol/L CsA was investigated in a collaboration between four European groups (Jennings et al. 2009). Changes in expression levels of the three biomarkers were not identified, suggesting alternative indicator genes should be considered if HK-2 cells are to be used for nephrotoxicity screening.

Compared with HK-2 cells, primary cells showed a much greater potential for establishing a system for in vitro drug-induced nephrotoxicity screening. Of the biomarkers assessed, protein levels in culture medium and cell lysates of KIM-1 and NGAL seemed to be more reliable than M-CSF. In the culture medium of hRPTECs, the concentrations of the three biomarkers were gradually increased with time (Fig. S9), with the longest incubation period (72 h) giving the greatest statistical significance. After 72 h treatment, the release of KIM-1 and NGAL protein in culture medium significantly increased after exposure to medium and high doses of all tested nephrotoxins, with clear dose-dependent responses (Fig.3A and C). M-CSF also showed increases; however, most compounds did not produce statistically significant effects (Fig.3E). In cell lysates, KIM-1 and NGAL proteins were also upregulated, with up to 20-fold increased NGAL protein levels at the 72-h time point (Fig.3B and D). An upregulation of mRNA levels of each biomarker was also observed in hRPTECs (Fig.4 and Fig. S8). High doses of CsA and doxorubicin resulted in significant induction of mRNA levels of all three biomarkers, with more variable results for the other compounds (Fig.4). Thus, mRNA levels may be less reliable in a screening assay than protein expression.

KIM-1 and NGAL were further investigated in hPT cells, which were freshly isolated from a human kidney. High doses of colistin, gentamicin, and cisplatin-induced significant upregulation of KIM-1 and NGAL proteins within 24 h (Fig.5). More than fivefold upregulation of KIM-1 protein expression and 60-fold increase in NGAL protein expression were observed in cell lysates, indicating a useful sensitivity of the two biomarkers for in vitro screening. In addition, we noticed that the expression levels of KIM-1 and NGAL in hPT control cells were much lower than seen in the control hRPTECs. This may be due to freeze-thaw cycles and passages of commercial hPRTECs, which could lead to cell injury and higher levels of biomarkers than would be present in in vivo cells. In contrast, the hPT cells are freshly prepared from kidneys, and they may express similar lower levels of biomarkers as in the in vivo environment.

M-CSF showed less value in predicting nephrotoxicity in vitro. M-CSF is mainly secreted into culture medium instead of accumulating in the cytoplasm and its concentration in hRPTEC lysates was around or less than 10 pg/mL in most cases, which was close to the detection limit of the ELISA kits. M-CSF protein levels in culture medium gradually increased over time, reaching >200 pg/mL at 72 h (Fig.3). As the nephrotoxin concentrations increased, a general trend toward increasing levels of M-CSF protein was observed; however, only a few were statistically significant. The mRNA levels of M-CSF (Fig.4) showed a similar pattern to KIM-1 and NGAL: a trend toward increased concentrations that was only statistically significant for some compounds tested.

A comparison of previously published in vivo data against our current in vitro assays for the upregulation of the three biomarkers by each nephrotoxin is summarized in Table2. A similar trend in KIM-1 and NGAL protein overexpression between in vivo and primary cell (hRPTEC and hPT)-based assays was observed; however, the in vivo data for M-CSF were only partially linked to the in vitro results. Variations in the extent of biomarker upregulation by the different compounds may reflect different mechanisms of toxicity.

**Table 2:** Comparison of published in vivo data and cell-based assays (72 h time point) in HK-2 cells, hRPTECs, and hPT cells

			Colistin	Gentamicin	Cisplatin	AmB
Upregulation of KIM-1	in vivo		N/A	>10-fold (Vaidya et al. 2010)	>5-fold (Vaidya et al. 2006)	N/A
	HK-2	P	−	−	−	−
		R	−	−	+	−
	hRPTECs	P	+	+	+	+
		R	−	−	−	−
	hPT	P(L)	>12-fold	Greater than fivefold	>32-fold	N/A
		R	N/A	N/A	N/A	N/A
Upregulation of NGAL	in vivo		(Eadon et al. 2013; Ghlissi et al. 2013)	Greater than twofold (Kai et al. 2013; Sun et al. 2013)	(Mishra et al. 2004; Paragas et al. 2011)	(Kondo et al. 2012)
	HK-2	P	−	−	−	−
		R	−	−	+	−
	hRPTECs	P	+	+	+	+
		R	+	−	+	−
	hPT	P(L)	>60-fold	>60-fold	>60-fold	N/A
		R	N/A	N/A	N/A	N/A
Upregulation of M-CSF	in vivo		N/A	+ (Razzaque and Taguchi 2002)	+ (Razzaque and Taguchi 2002; Menke et al. 2009)	N/A
	HK-2	P	−	−	−	−
		R	−	−	+	−
	hRPTECs	P	−	+	+	−
		R	+	−	+	−

N/A, not applicable; P, protein levels in culture medium; P(L), protein levels in cell lysates; R, mRNA levels; +, statistically significant increase; −, no significant increase.

In summary, we evaluated in vitro changes in expression of three novel kidney injury biomarkers in HK-2 cells, hRPTECs and hPT cells upon exposure to known nephrotoxic compounds. Although the superiority of primary cultures for predicting nephrotoxicity may be known to an expert reviewer, there is no published systematic study regarding the expression changes of in vivo biomarker in either immortalized or primary cells, in response to exposure to nephrotoxic drugs and drug candidates. Our data provide the first rigorous confirmation that the primary cell line cultures are clearly superior, particularly for relatively new biomarkers that are becoming increasingly important for in vivo diagnosis. Significant increases in KIM-1 and NGAL protein were observed for all the six nephrotoxic compounds in the primary cell cultures, establishing that this combination has potential as an in vitro predictive screening system. Mechanisms of drug-induced nephrotoxicity in vivo are complex, and some may involve flow-related absorption of drugs leading to high, localized concentrations at cell-structure interfaces that are difficult to replicate in an in vitro environment. Nonetheless, assays that are predictive of at least some compound-induced mechanisms leading to renal damage will have great utility in providing early feedback to minimize these liabilities during the development of new drugs.

## Abbreviations

AKI: acute kidney injury
BUN: blood urea nitrogen
CC_50_: the concentration of 50% cellular toxicity
CKD: chronic kidney disease
DMEM/F12: Dulbecco’s Modified Eagle’s Medium: Ham’s F12 Medium
HPRT1: hypoxanthine phosphoribosyltransferase 1
hPT: human proximal tubular
hRPTECs: primary human renal proximal tubule epithelial cells
KIM-1/TIM-1/HAVCR1: kidney injury molecule-1
LDH: lactate dehydrogenase
M-CSF/CSF-1: human macrophage colony stimulating factor
MTT: 3-(4,5-dimethylthiazol-2-yl)-2,5-diphenyltetrazolium bromide
NGAL/LCN2: neutrophil gelatinase-associated lipocalin
qRT-PCR: real-time quantitative reverse transcription PCR
SCr: serum creatinine

## Supporting Information

**Figure S1.** Expression profile of biomarkers in HK2 cells after nephrotoxic compound treatment for 4 h. (A) KIM-1 protein concentration in culture medium; (B) KIM-1 protein concentration in cell lysates; (C) NGAL protein concentration in culture medium; (D) NGAL protein concentration in cell lysates; (E) M-CSF protein concentration in culture medium; and (F) M-CSF protein concentration in cell lysates. Data are presented as mean ± SD, *n *≥ 3.

**Figure S2.** Expression profile of biomarkers in HK2 cells after nephrotoxic compound treatment for 24 h. (A) KIM-1 protein concentration in culture medium; (B) KIM-1 protein concentration in cell lysates; (C) NGAL protein concentration in culture medium; (D) NGAL protein concentration in cell lysates; (E) M-CSF protein concentration in culture medium; and (F) M-CSF protein concentration in cell lysates. Data are presented as mean ± sd. Significantly different ***P *< 0.01; *n *≥ 3.

**Figure S3.** Expression profile of biomarkers in HK2 cells after nephrotoxic compound treatment for 48 h. (A) KIM-1 protein concentration in culture medium; (B) KIM-1 protein concentration in cell lysates; (C) NGAL protein concentration in culture medium; (D) NGAL protein concentration in cell lysates; (E) M-CSF protein concentration in culture medium; and (F) M-CSF protein concentration in cell lysates. Data are presented as mean ± SD. Significantly different **P *< 0.05; *n *≥ 3.

**Figure S4.** mRNA levels of each biomarkers in HK2 cells after nephrotoxic compound treatment for 4, 24 and 48 h. (A, B and C) KIM-1 mRNA, (D, E and F) NGAL mRNA and (G, H and I) M-CSF mRNA. Data are presented as mean ± sd. Significantly different **P *< 0.05; ***P *< 0.01; ****P *< 0.005, *n *≥ 3.

**Figure S5.** Expression profile of biomarkers in hRPTEC cells after nephrotoxic compound treatment for 4 h. (A) KIM-1 protein concentration in culture medium; (B) KIM-1 protein concentration in cell lysates; (C) NGAL protein concentration in culture medium; (D) NGAL protein concentration in cell lysates; (E) M-CSF protein concentration in culture medium; and (F) M-CSF protein concentration in cell lysates. Data are presented as mean ± SD. Significantly different **P *< 0.05, *n *≥ 3.

**Figure S6.** Expression profile of biomarkers in hRPTEC cells after nephrotoxic compound treatment for 24 h. (A) KIM-1 protein concentration in culture medium; (B) KIM-1 protein concentration in cell lysates; (C) NGAL protein concentration in culture medium; (D) NGAL protein concentration in cell lysates; (E) M-CSF protein concentration in culture medium; and (F) M-CSF protein concentration in cell lysates. Data are presented as mean ± sd. Significantly different **P *< 0.05, ***P *< 0.01; ****P *< 0.005; *****P *< 0.001, *n *≥ 3.

**Figure S7.** Expression profile of biomarkers in hRPTEC cells after nephrotoxic compound treatment for 48 h. (A) KIM-1 protein concentration in culture medium; (B) KIM-1 protein concentration in cell lysates; (C) NGAL protein concentration in culture medium; (D) NGAL protein concentration in cell lysates; (E) M-CSF protein concentration in culture medium; and (F) M-CSF protein concentration in cell lysates. Data are presented as mean ± SD. Significantly different * *P *< 0.05, ***P *< 0.01; ****P *< 0.005; *****P *< 0.001, *n *≥ 3.

**Figure S8.**mRNA levels of each biomarkers in hRPTEC cells after nephrotoxic compounds treatment for 4, 24 and 48 h. (A, B and C) KIM-1 mRNA, (D, E and F) NGAL mRNA and (G, H and I) M-CSF mRNA. Data are presented as mean ± sd. Significantly different **P *< 0.05; ***P *< 0.01; ****P *< 0.005, *****P *< 0.001, *n *≥ 3.

**Figure S9.** An example of time-dependent increases of biomarker protein levels in hRPTEC culture medium. Dot lines, culture medium of untreated control cells; Solid lines, culture medium of 20 *µ*mol/L CsA treated cells. Round solid dots, KIM-1; Triangles, NGAL and squares, M-CSF. Data are presented as mean ± SD.
