# Supplementary figures and images for: Evaluation of biomarkers for in vitro prediction of drug-induced nephrotoxicity: comparison of HK-2, immortalized human proximal tubule epithelial, and primary cultures of human proximal tubular cells
Source: Pharmacol Res Perspect. 2015 May 15;3(3):e00148. doi: 10.1002/prp2.148 (PMC4492764; doi:10.1002/prp2.148)

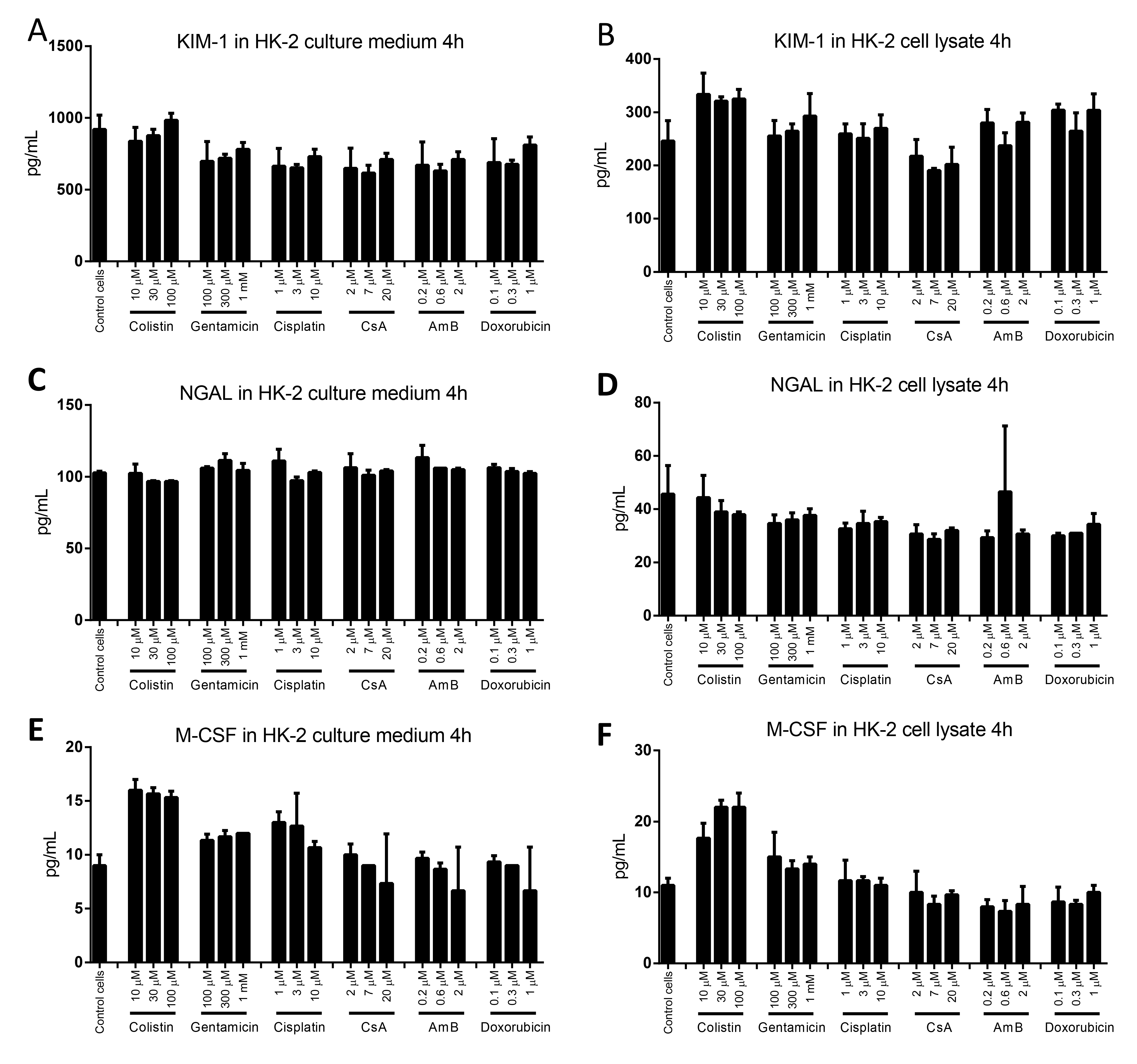

Supplement: Supplementary file 1 [file prp20003-e00148-sd1.tif]

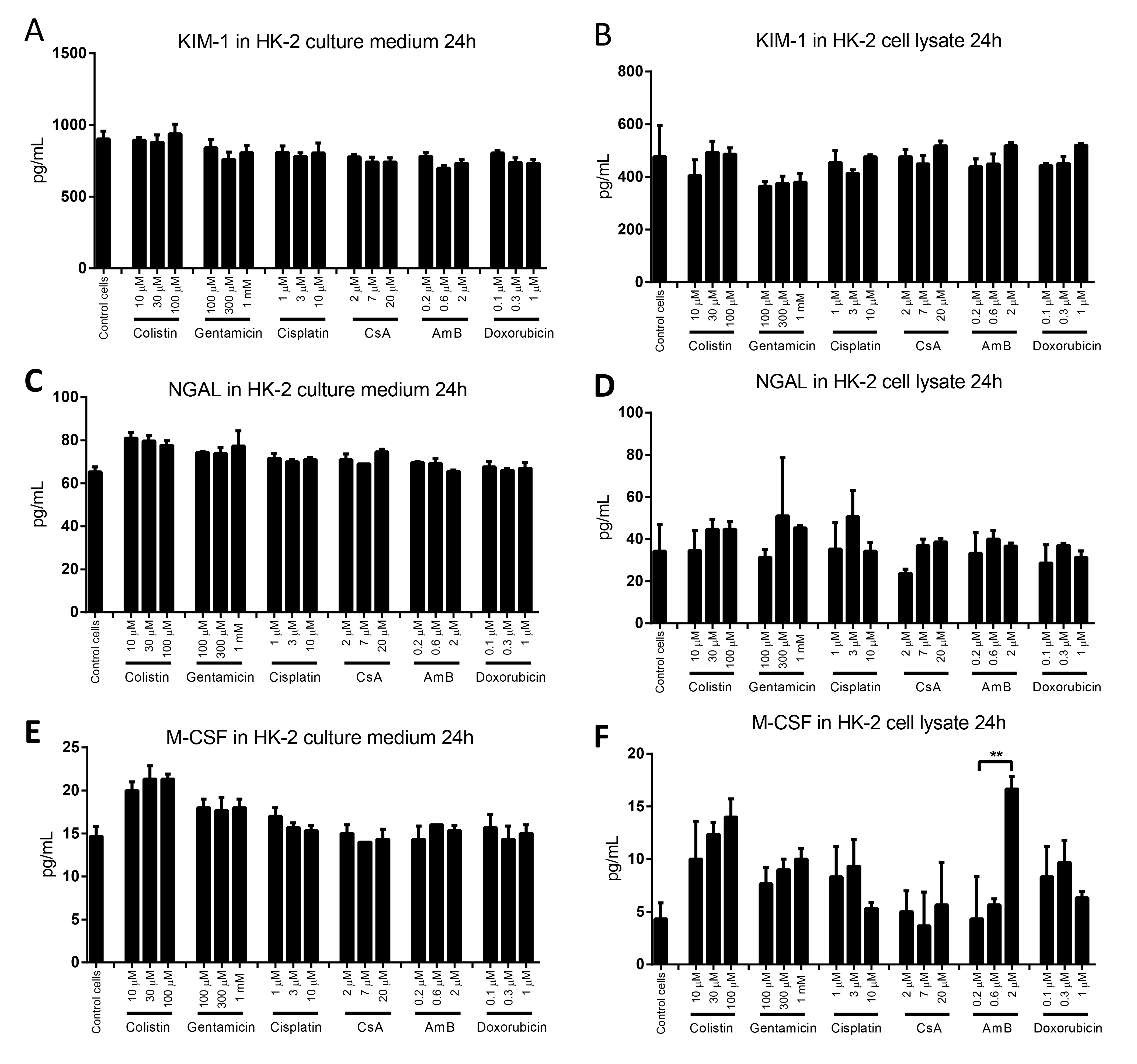

Supplement: Supplementary file 2 [file prp20003-e00148-sd2.tif]

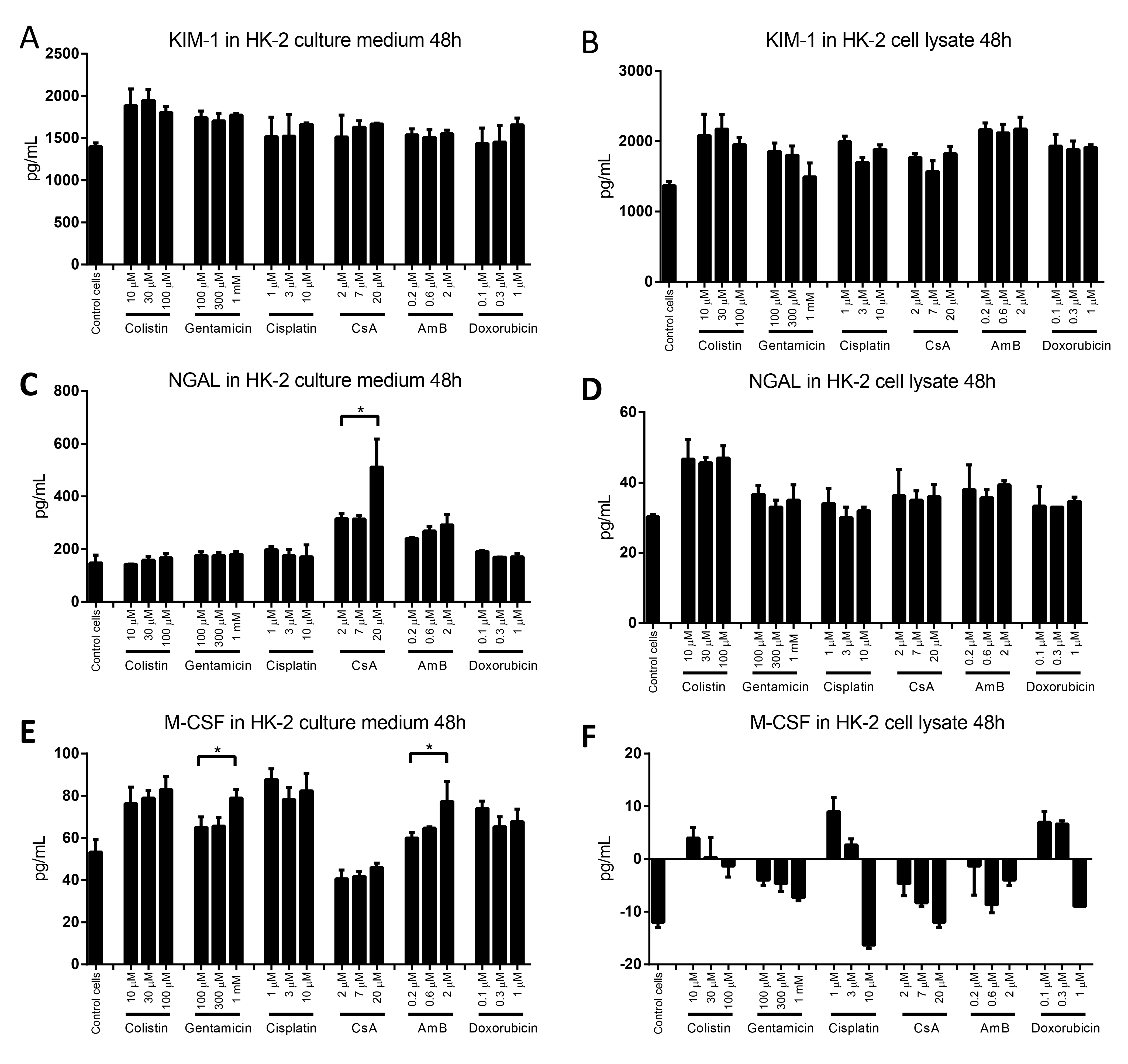

Supplement: Supplementary file 3 [file prp20003-e00148-sd3.tif]

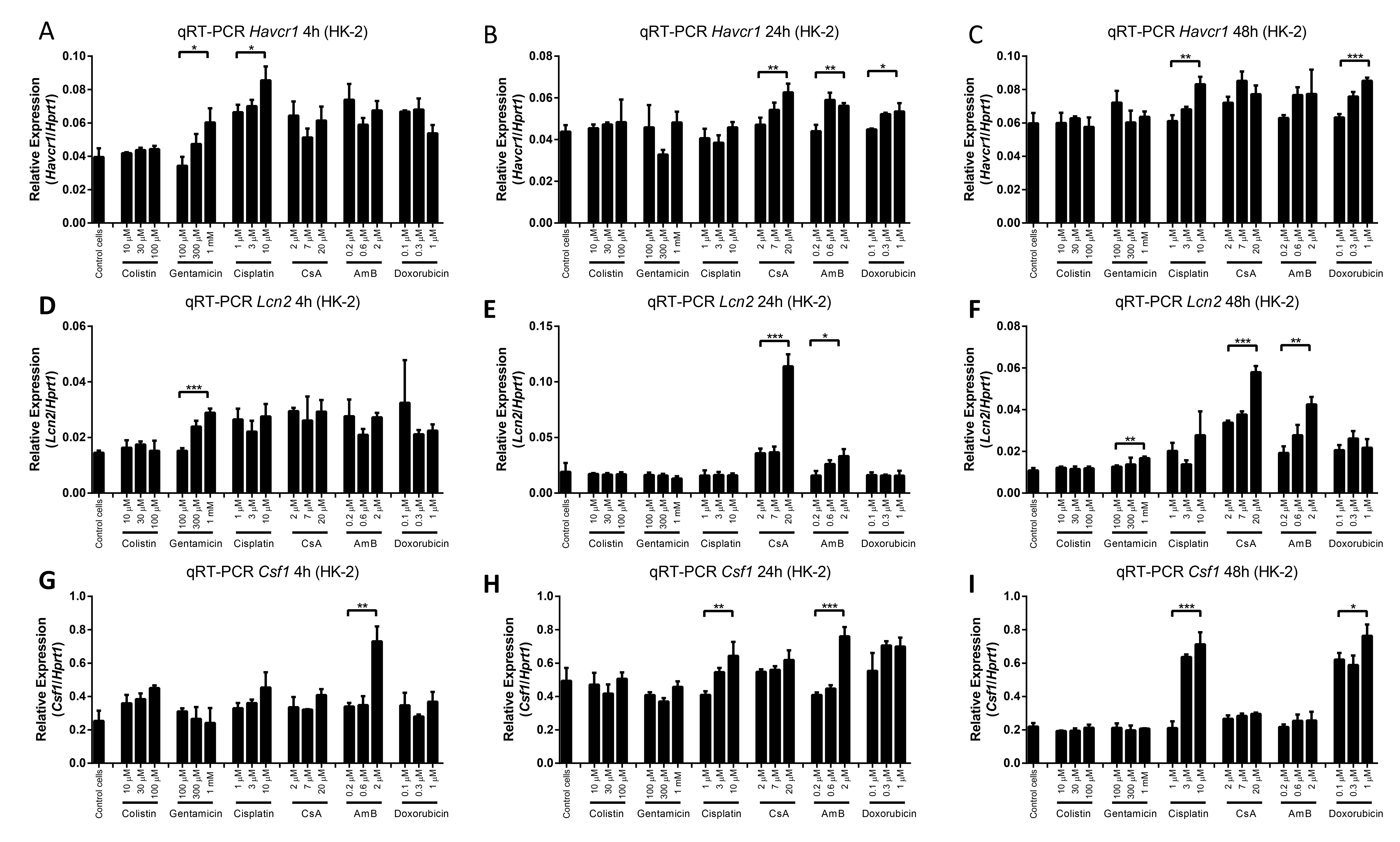

Supplement: Supplementary file 4 [file prp20003-e00148-sd4.tif]

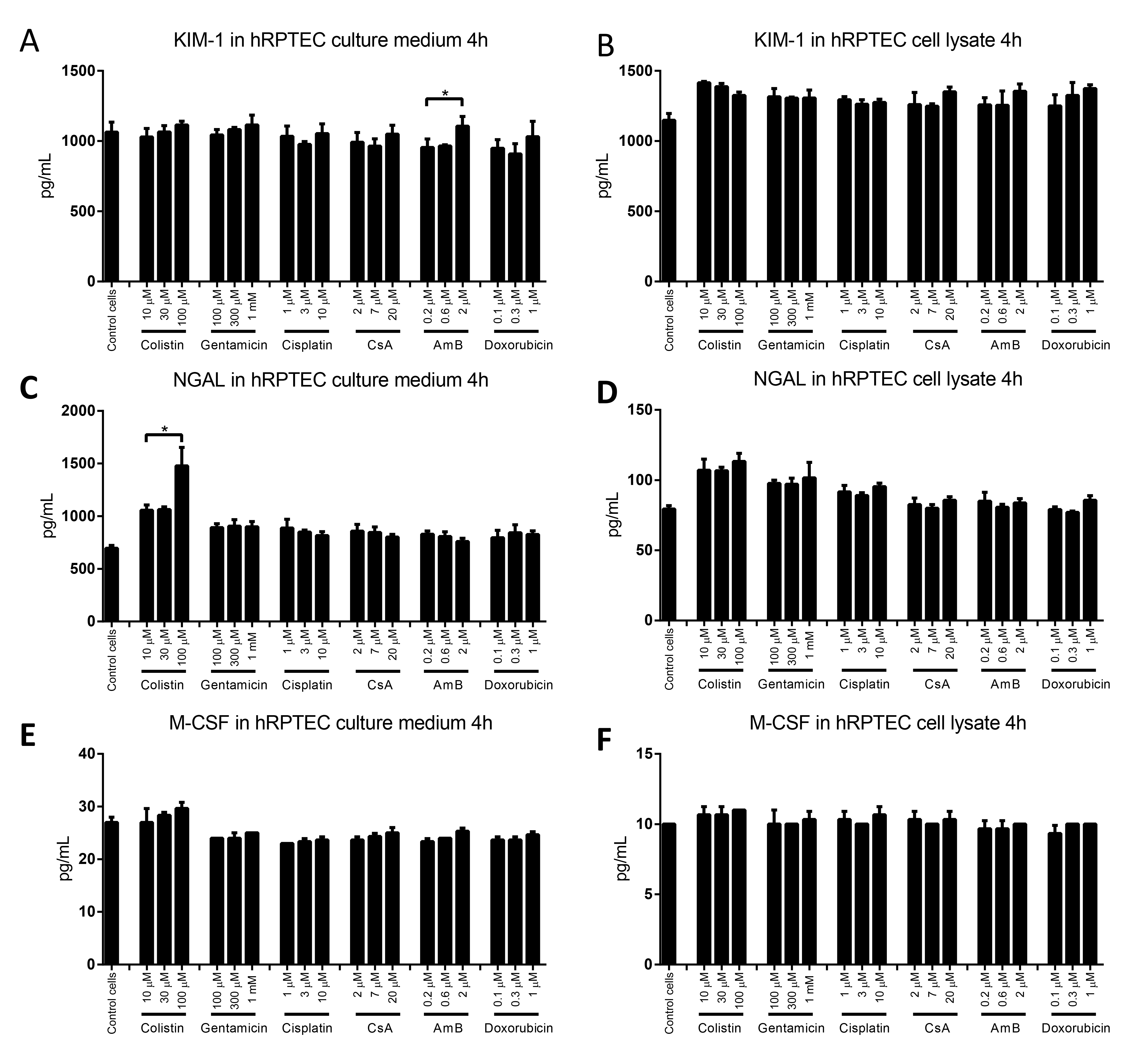

Supplement: Supplementary file 5 [file prp20003-e00148-sd5.tif]

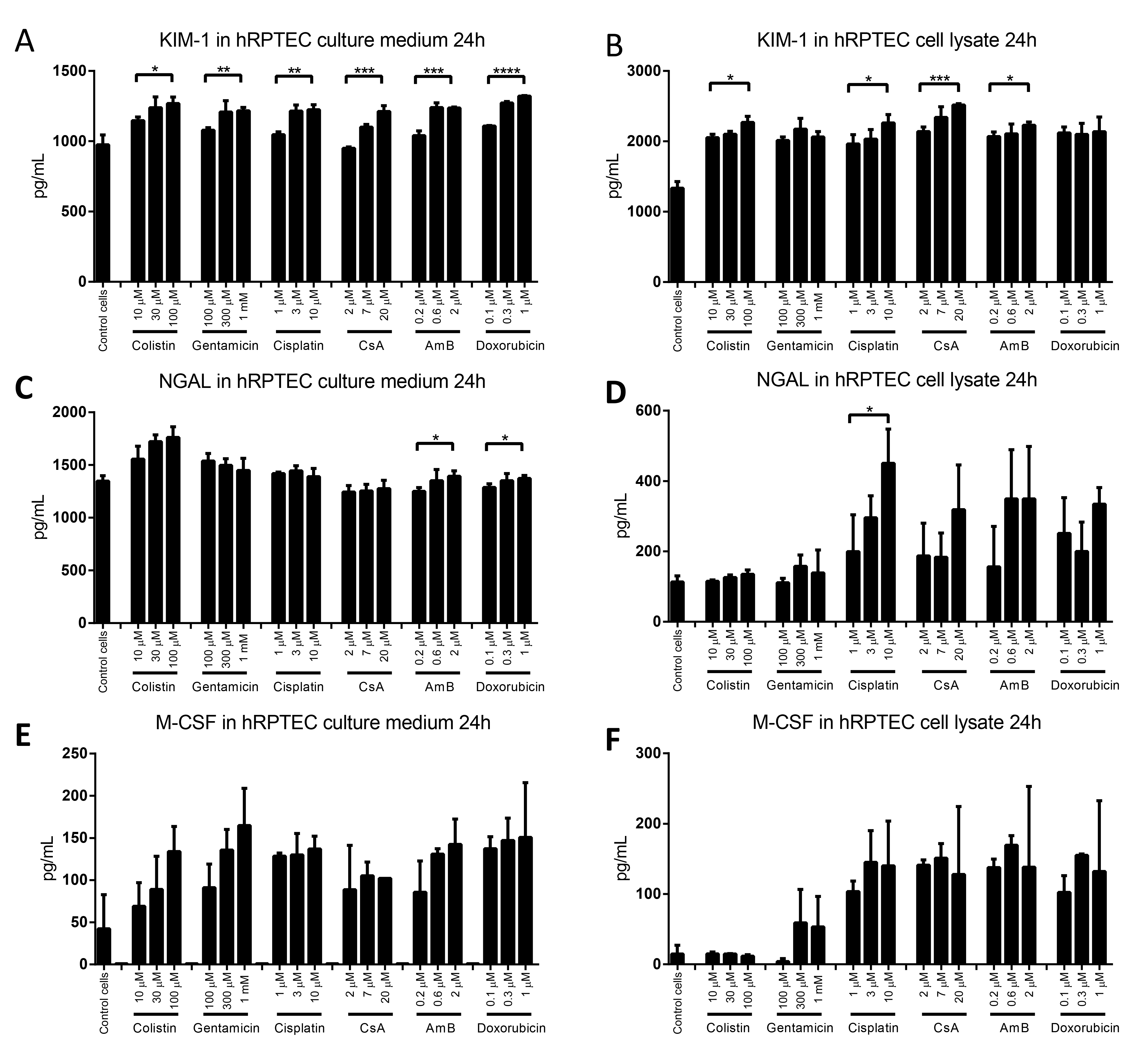

Supplement: Supplementary file 6 [file prp20003-e00148-sd6.tif]

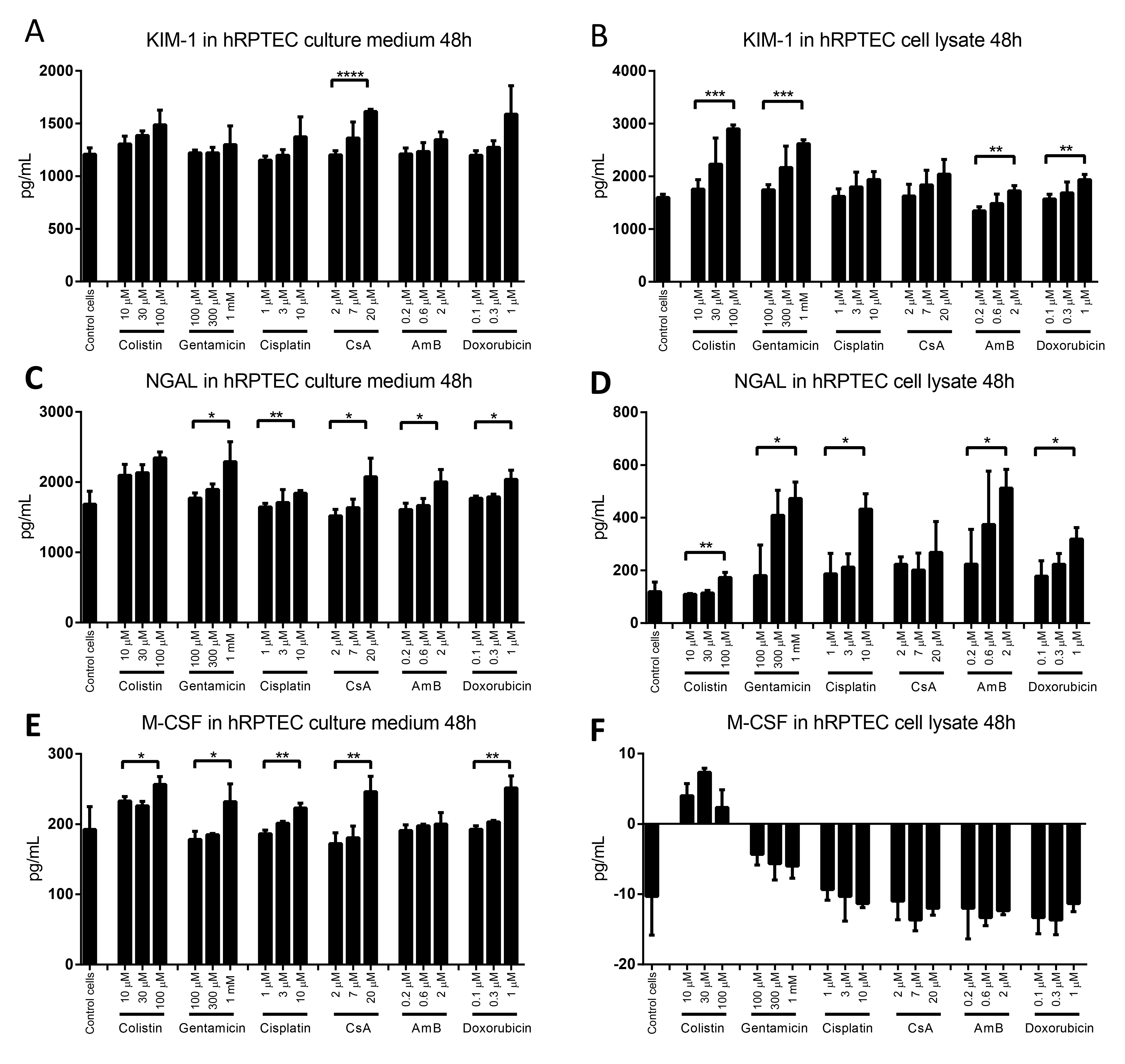

Supplement: Supplementary file 7 [file prp20003-e00148-sd7.tif]

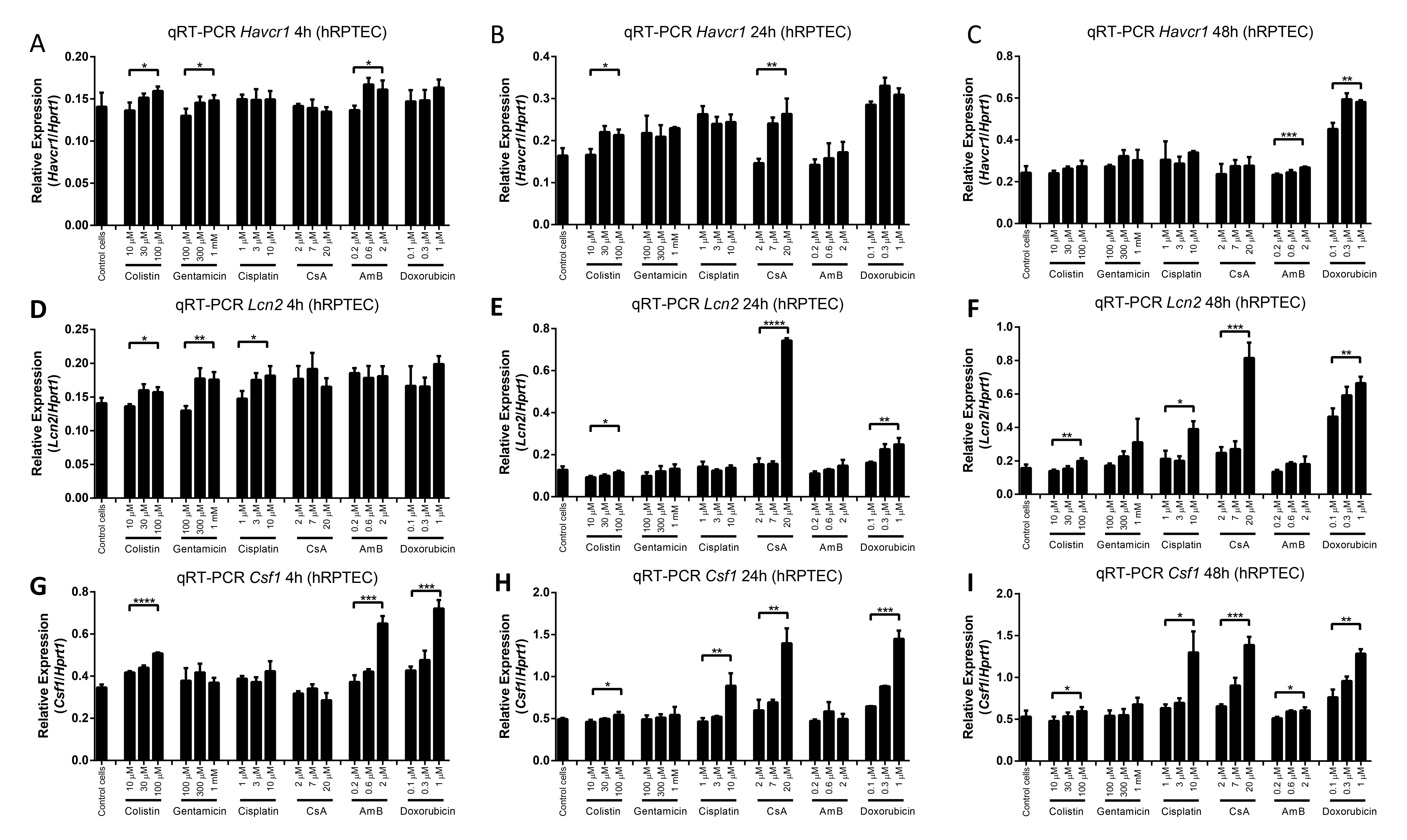

Supplement: Supplementary file 8 [file prp20003-e00148-sd8.tif]
