# Supplementary material for: Evaluation of biomarkers for in vitro prediction of drug-induced nephrotoxicity: comparison of HK-2, immortalized human proximal tubule epithelial, and primary cultures of human proximal tubular cells
Source: Pharmacol Res Perspect. 2015 May 15;3(3):e00148. doi: 10.1002/prp2.148 (PMC4492764; doi:10.1002/prp2.148)
Supplement: Supplementary file 10 [file prp20003-e00148-sd10.docx]

**Appendix (Supporting information)**

**Evaluation of biomarkers for *in vitro* prediction of drug-induced nephrotoxicity: comparison of HK-2, immortalized human proximal tubule epithelial, and primary cultures of human proximal tubular cells**

Johnny X. Huang^1^, Geraldine Kaeslin^1^, Max V. Ranall^1^, Mark A. Blaskovich^1^, Bernd Becker^1^, Mark S. Butler^1^, Melissa H. Little^1^, Lawrence H. Lash^2^ and Matthew A. Cooper^1^

1. *Institute for Molecular Bioscience, The University of Queensland, 306 Carmody Road, St Lucia, QLD 4072, Australia.*
2. *Department of Pharmacology, Wayne State University, School of Medicine, 540 East Canfield Avenue, Detroit, MI 48201 USA*


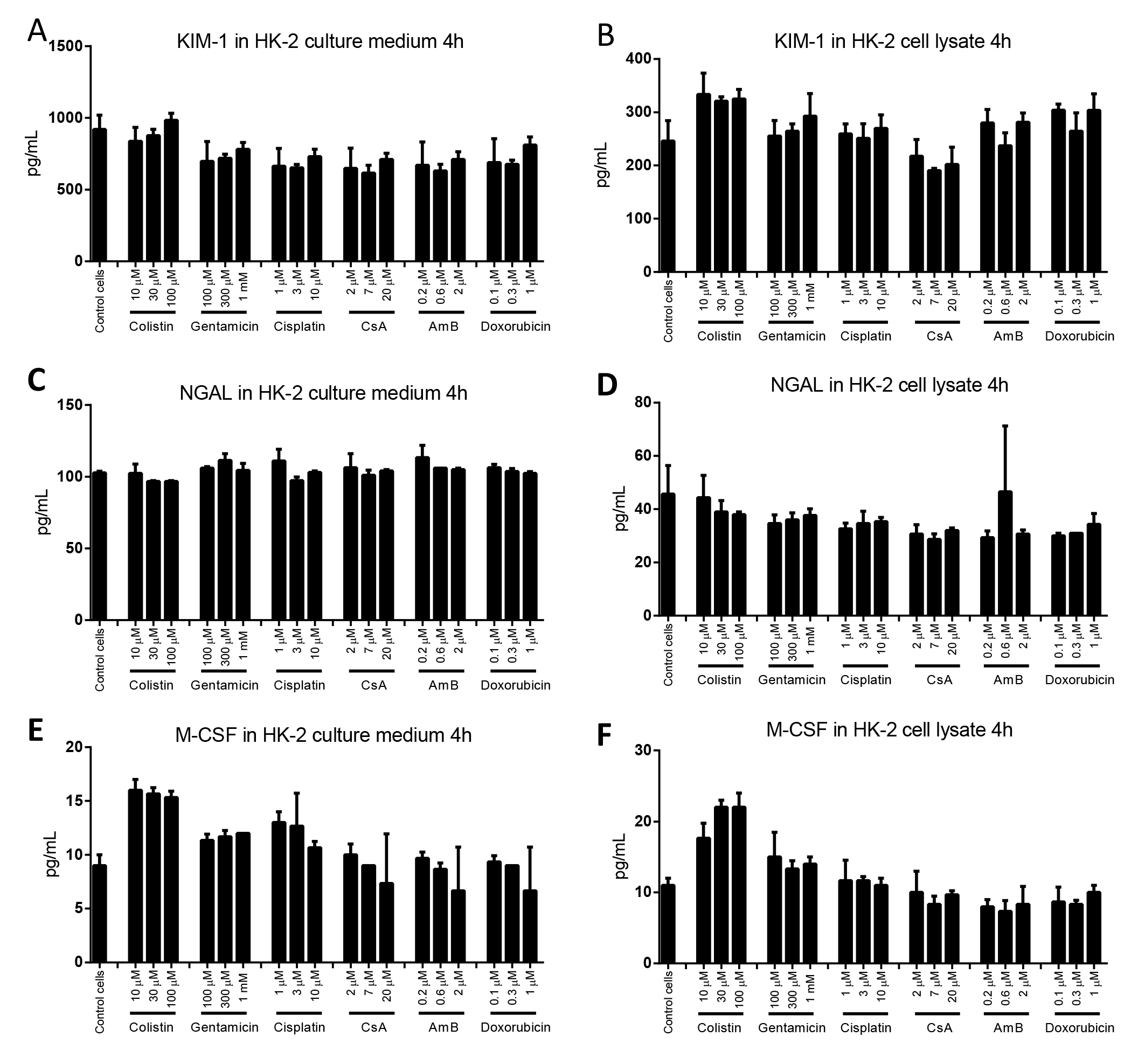


Fig. S1. Expression profile of biomarkers in HK2 cells after nephrotoxic compound treatment for 4 h. (A) KIM-1 protein concentration in culture medium; (B) KIM-1 protein concentration in cell lysates; (C) NGAL protein concentration in culture medium; (D) NGAL protein concentration in cell lysates; (E) M-CSF protein concentration in culture medium; and (F) M-CSF protein concentration in cell lysates. Data are presented as Mean ± Standard deviation. *n*≥3.


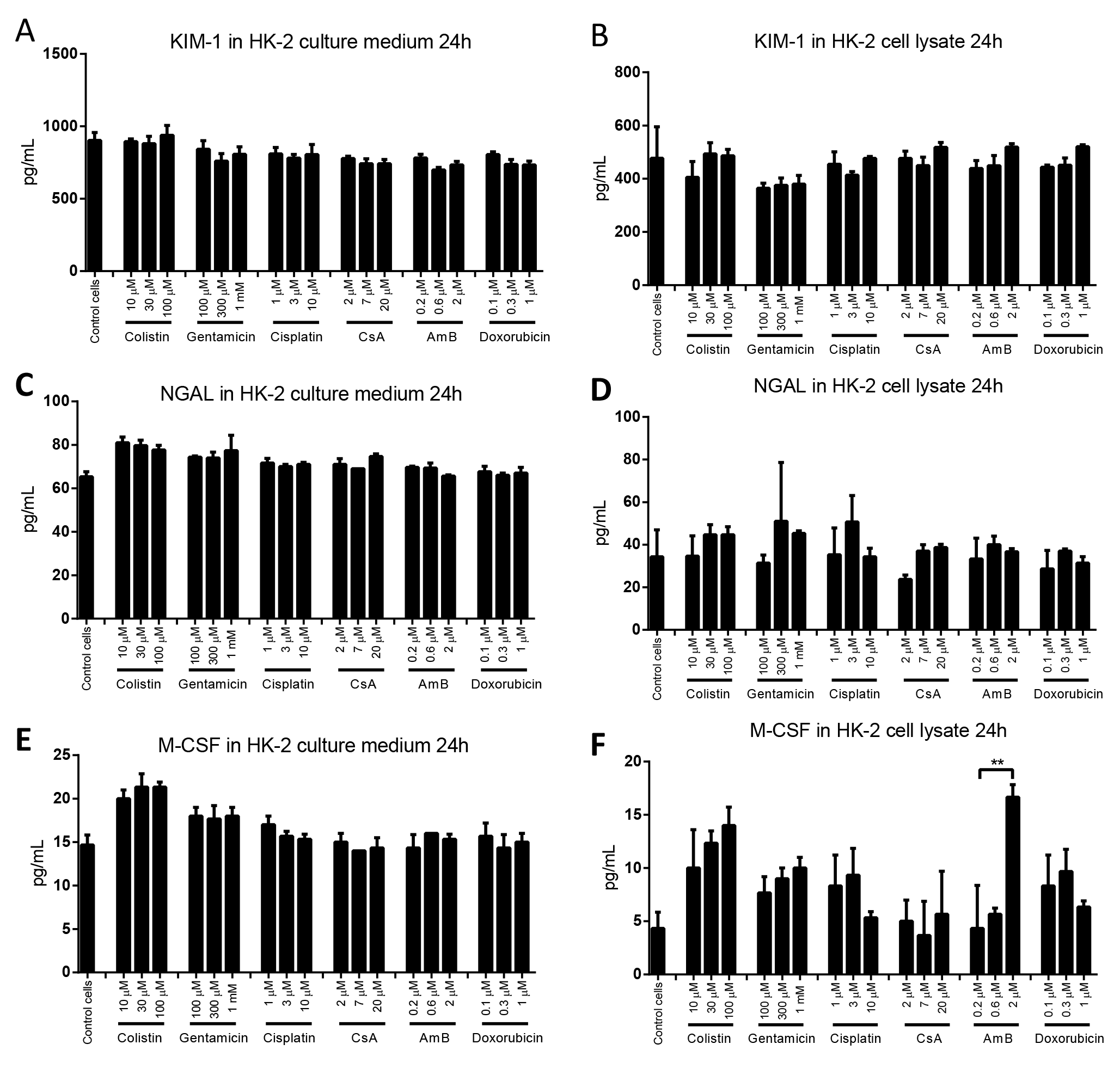


Fig. S2. Expression profile of biomarkers in HK2 cells after nephrotoxic compound treatment for 24 h. (A) KIM-1 protein concentration in culture medium; (B) KIM-1 protein concentration in cell lysates; (C) NGAL protein concentration in culture medium; (D) NGAL protein concentration in cell lysates; (E) M-CSF protein concentration in culture medium; and (F) M-CSF protein concentration in cell lysates. Data are presented as Mean ± Standard deviation. Significantly different ***P*<0.01; *n*≥3.


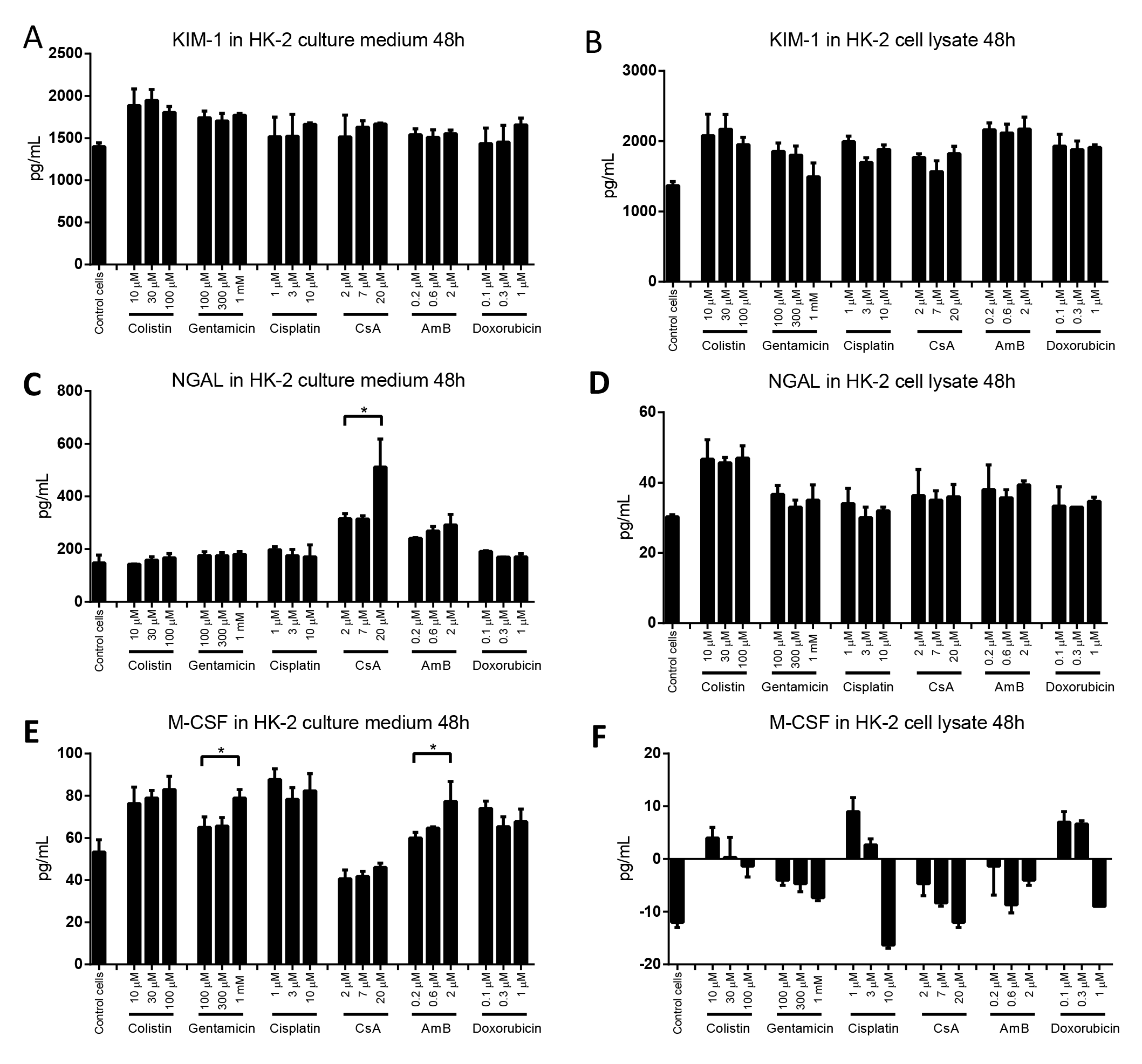


Fig. S3. Expression profile of biomarkers in HK2 cells after nephrotoxic compound treatment for 48 h. (A) KIM-1 protein concentration in culture medium; (B) KIM-1 protein concentration in cell lysates; (C) NGAL protein concentration in culture medium; (D) NGAL protein concentration in cell lysates; (E) M-CSF protein concentration in culture medium; and (F) M-CSF protein concentration in cell lysates. Data are presented as Mean ± Standard deviation. Significantly different **P*<0.05; *n*≥3.


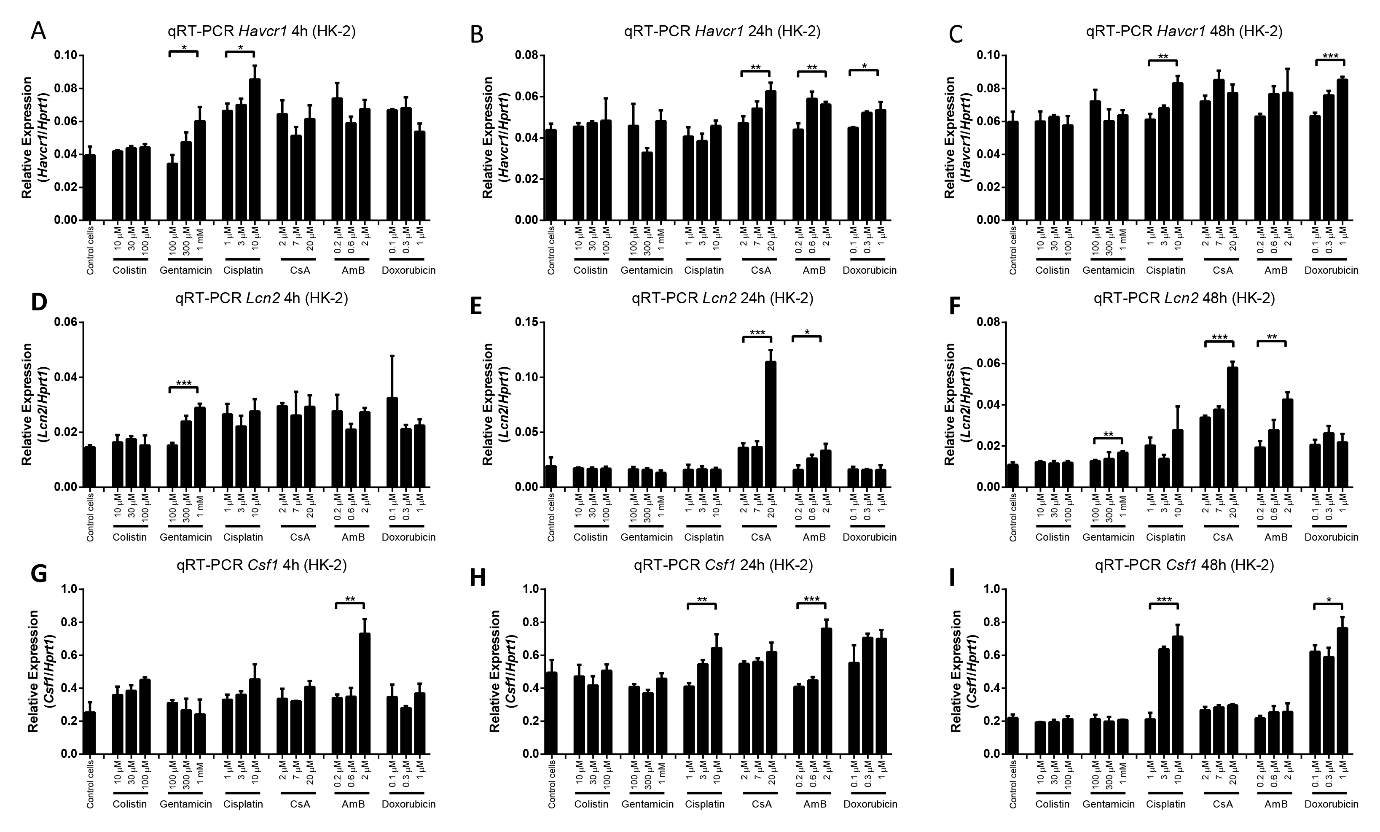


Fig. S4. mRNA levels of each biomarkers in HK2 cells after nephrotoxic compound treatment for 4, 24 and 48 h. (A, B and C) KIM-1 mRNA, (D, E and F) NGAL mRNA and (G, H and I) M-CSF mRNA. Data are presented as Mean ± Standard deviation. Significantly different * *P*<0.05; ***P*<0.01; ****P*<0.005, *n*≥3.


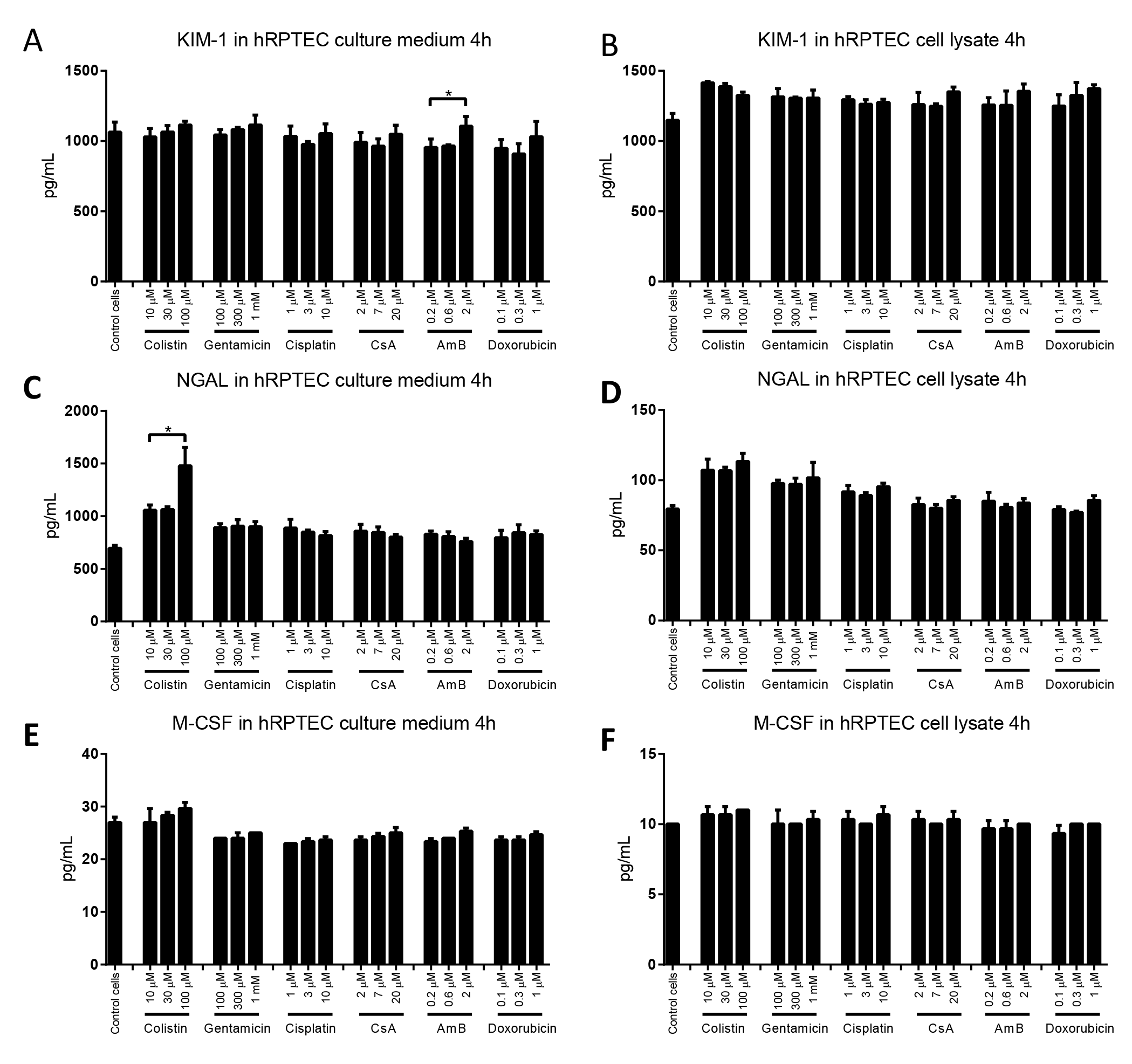


Fig. S5. Expression profile of biomarkers in hRPTEC cells after nephrotoxic compound treatment for 4 h. (A) KIM-1 protein concentration in culture medium; (B) KIM-1 protein concentration in cell lysates; (C) NGAL protein concentration in culture medium; (D) NGAL protein concentration in cell lysates; (E) M-CSF protein concentration in culture medium; and (F) M-CSF protein concentration in cell lysates. Data are presented as Mean ± Standard deviation. Significantly different * *P*<0.05, *n*≥3.


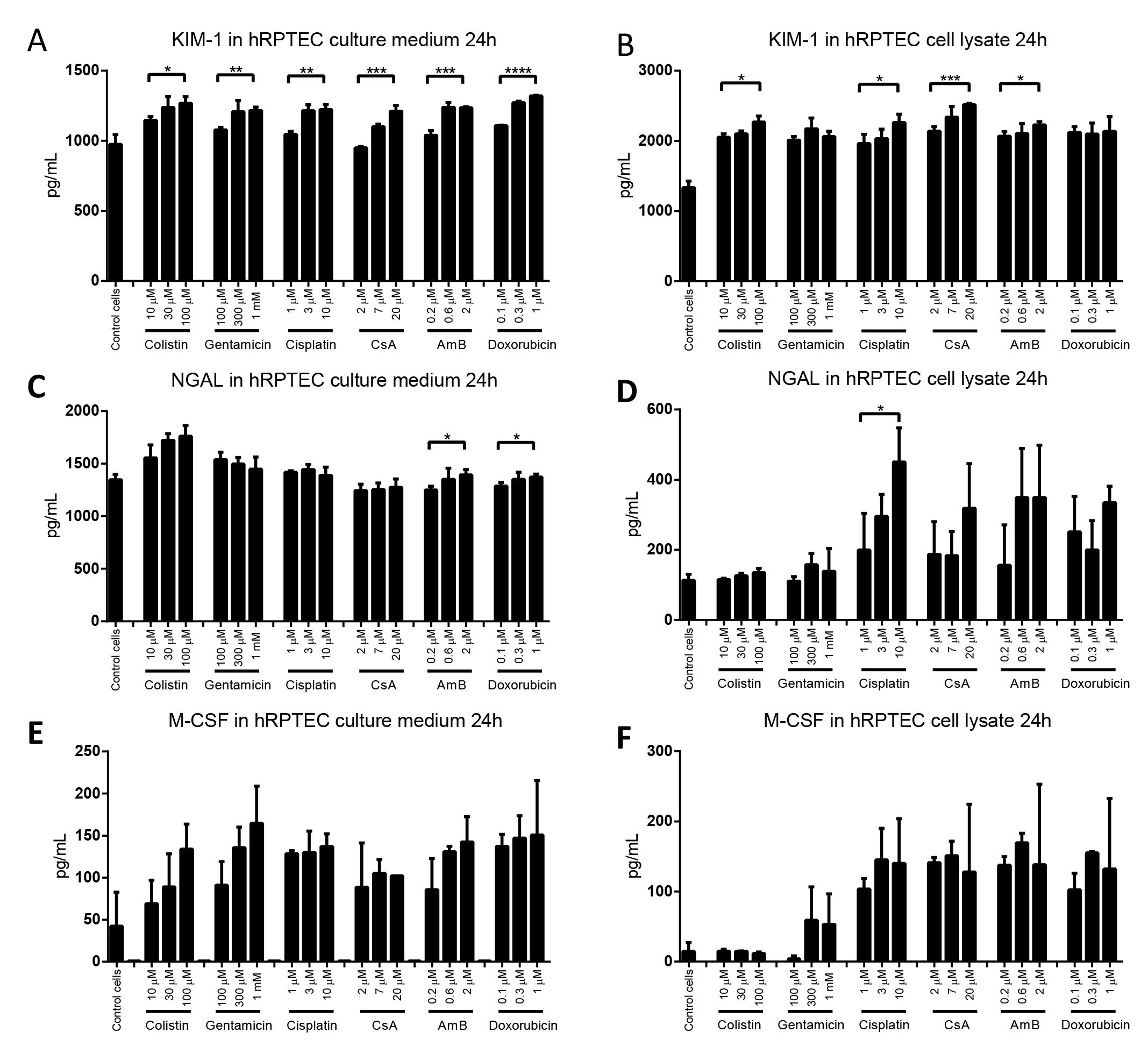


Fig. S6. Expression profile of biomarkers in hRPTEC cells after nephrotoxic compound treatment for 24 h. (A) KIM-1 protein concentration in culture medium; (B) KIM-1 protein concentration in cell lysates; (C) NGAL protein concentration in culture medium; (D) NGAL protein concentration in cell lysates; (E) M-CSF protein concentration in culture medium; and (F) M-CSF protein concentration in cell lysates. Data are presented as Mean ± Standard deviation. Significantly different * *P*<0.05, ***P*<0.01; ****P*<0.005; **** *P*<0.001, *n*≥3.


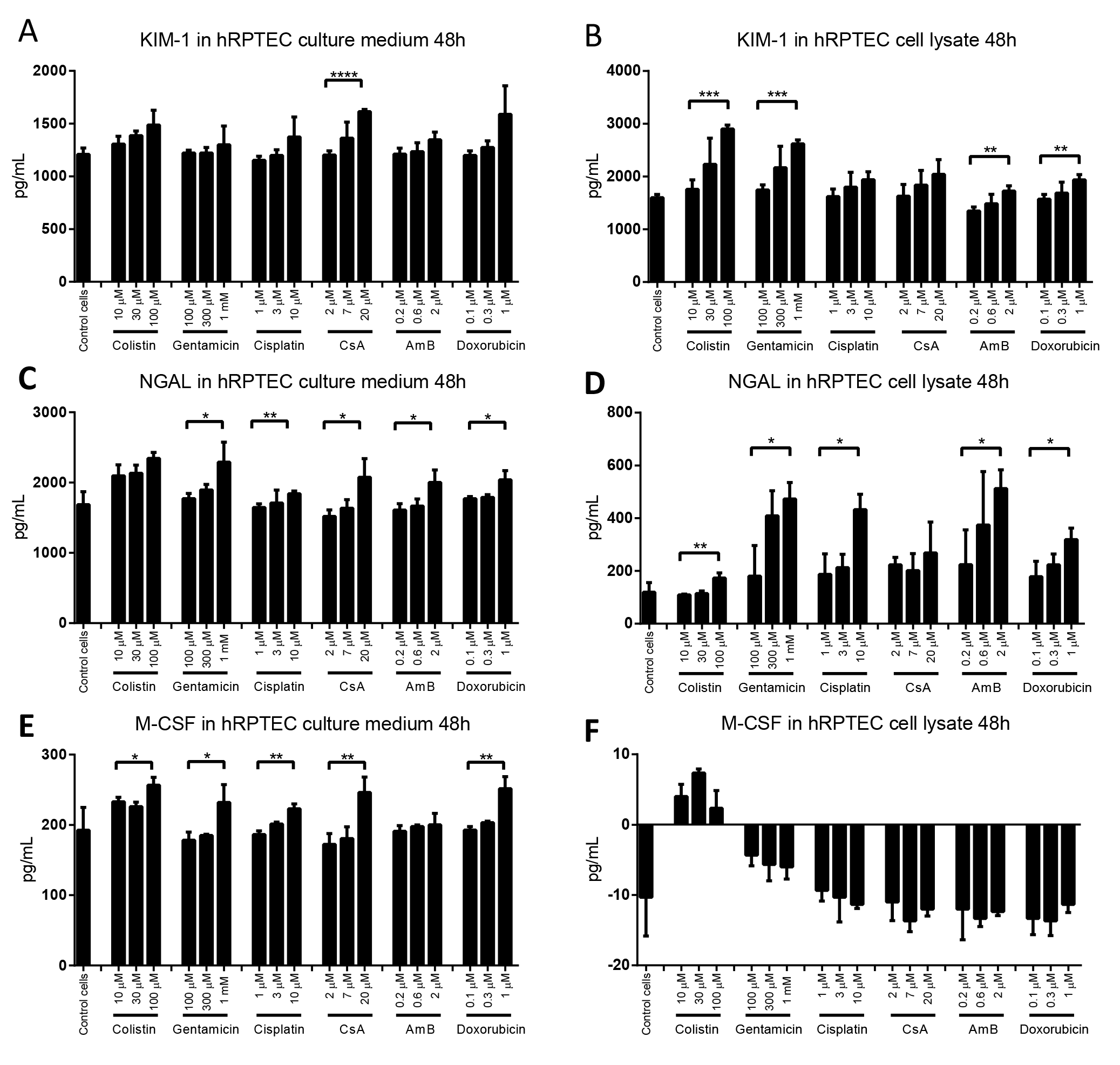


Fig. S7. Expression profile of biomarkers in hRPTEC cells after nephrotoxic compound treatment for 48 h. (A) KIM-1 protein concentration in culture medium; (B) KIM-1 protein concentration in cell lysates; (C) NGAL protein concentration in culture medium; (D) NGAL protein concentration in cell lysates; (E) M-CSF protein concentration in culture medium; and (F) M-CSF protein concentration in cell lysates. Data are presented as Mean ± Standard deviation. Significantly different * *P*<0.05, ***P*<0.01; ****P*<0.005; *****P*<0.001, *n*≥3.


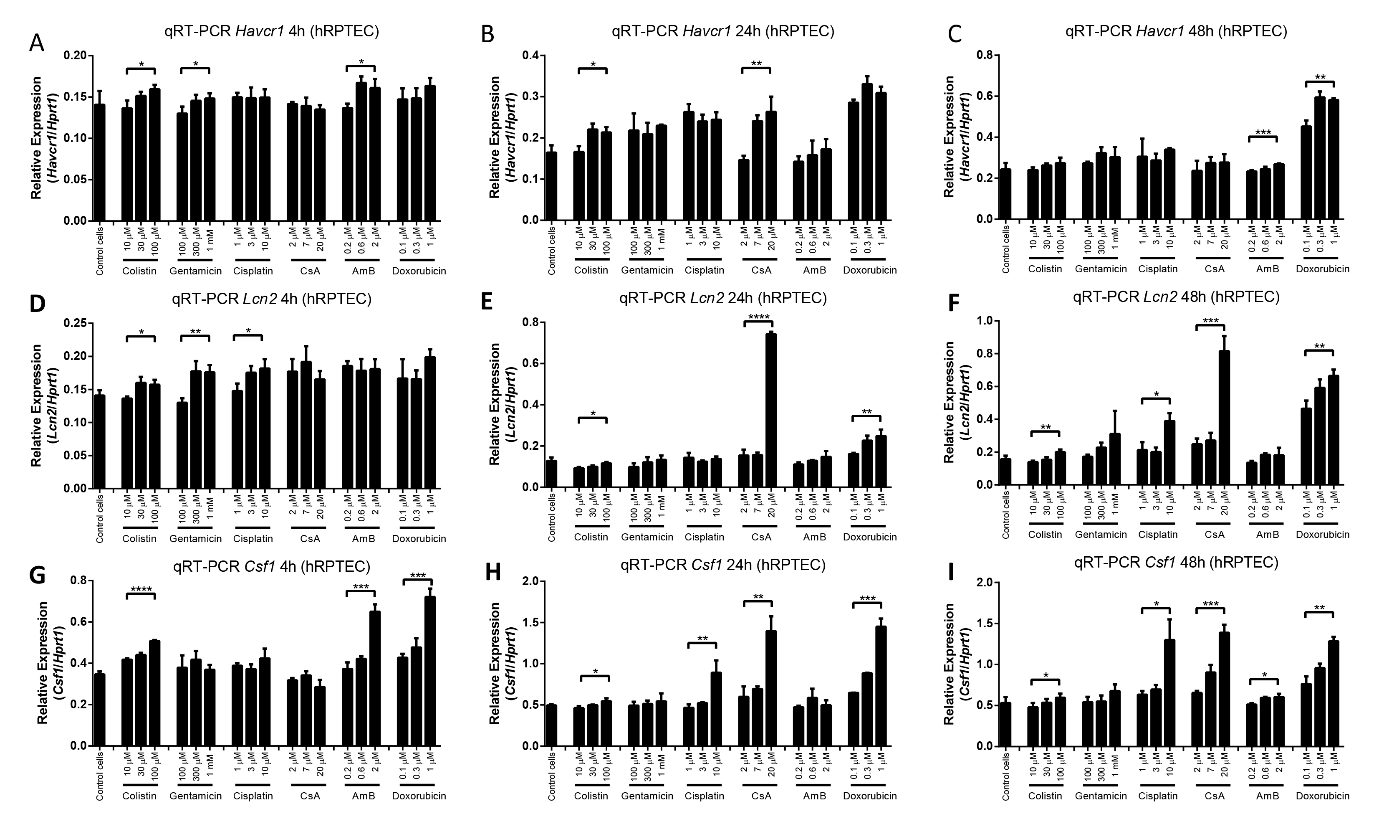


Fig. S8. mRNA levels of each biomarkers in hRPTEC cells after nephrotoxic compounds treatment for 4, 24 and 48 h. (A, B and C) KIM-1 mRNA, (D, E and F) NGAL mRNA and (G, H and I) M-CSF mRNA. Data are presented as Mean ± Standard deviation. Significantly different * *P*<0.05; ***P*<0.01; ****P*<0.005, *****P*<0.001, *n*≥3.


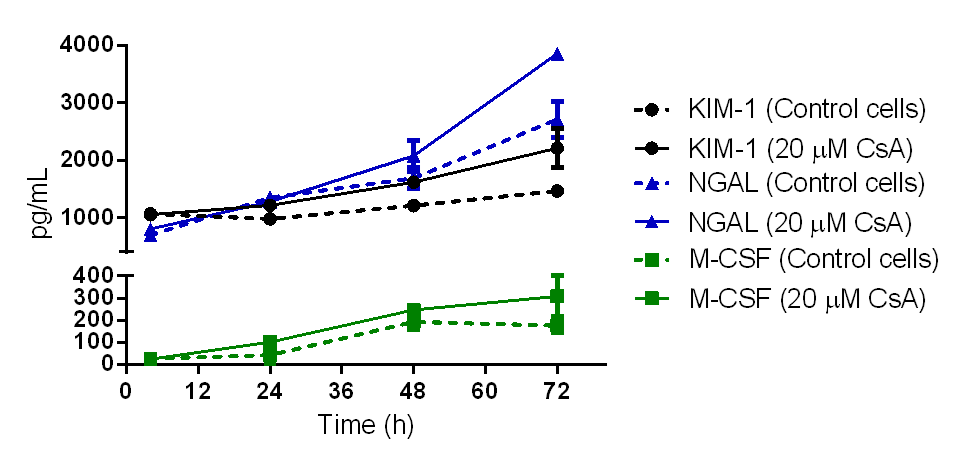


Fig. S9. An example of time-dependent increases of biomarker protein levels in hRPTEC culture medium. Dot lines, culture medium of untreated control cells; Solid lines, culture medium of 20 µM CsA treated cells. Round solid dots, KIM-1; Triangles, NGAL and Squares, M-CSF. Data are presented as Mean ± Standard deviation.
